# GPU-accelerated Kendall distance computation for large or sparse data

**DOI:** 10.1093/gigascience/giae088

**Published:** 2024-12-09

**Authors:** Pavel Akhtyamov, Ausaaf Nabi, Vladislav Gafurov, Alexey Sizykh, Alexander Favorov, Yulia Medvedeva, Alexey Stupnikov

**Affiliations:** Department of Biomedical Physics, Moscow Institute of Physics and Technology, 141701, Dolgoprudny, Russia; Moscow Center for Advanced Studies, Department of Biomedical Physics, Moscow, 123592, Russia; Department of Biomedical Physics, Moscow Institute of Physics and Technology, 141701, Dolgoprudny, Russia; Moscow Center for Advanced Studies, Department of Biomedical Physics, Moscow, 123592, Russia; Department of Biomedical Physics, Moscow Institute of Physics and Technology, 141701, Dolgoprudny, Russia; Moscow Center for Advanced Studies, Department of Biomedical Physics, Moscow, 123592, Russia; Department of Biomedical Physics, Moscow Institute of Physics and Technology, 141701, Dolgoprudny, Russia; Moscow Center for Advanced Studies, Department of Biomedical Physics, Moscow, 123592, Russia; University of Manitoba, Department of Biochemistry and Medical Genetics, Winnipeg, MB R3E 3P5, Canada; Johns Hopkins University School of Medicine, Department of Oncology, Sidney Kimmel Comprehensive Cancer Center, Baltimore, MD 21205, USA; Vavilov Institute of General Genetics, Laboratory of Systems Biology and Computational Genetics, Moscow, 119333, Russia; Department of Biomedical Physics, Moscow Institute of Physics and Technology, 141701, Dolgoprudny, Russia; Moscow Center for Advanced Studies, Department of Biomedical Physics, Moscow, 123592, Russia; Research Center of Biotechnology, Institute of Bioengineering, 117312, Moscow, Russia; Department of Biomedical Physics, Moscow Institute of Physics and Technology, 141701, Dolgoprudny, Russia; Moscow Center for Advanced Studies, Department of Biomedical Physics, Moscow, 123592, Russia

**Keywords:** Kendall correlation, distance matrix, GPU, parallel computation, high dimension, scRNA-seq, scATAC-seq

## Abstract

**Background:**

Current experimental practices typically produce large multidimensional datasets. Distance matrix calculation between elements (e.g., samples) for such data, although being often necessary in preprocessing for statistical inference or visualization, can be computationally demanding. Data sparsity, which is often observed in various experimental data modalities, such as single-cell sequencing in bioinformatics or collaborative filtering in recommendation systems, may pose additional algorithmic challenges.

**Results:**

We present GPU-Assisted Distance Estimation Software (GADES), a graphical processing unit (GPU)–enhanced package that allows for massively paralleled Kendall-$\tau$ distance matrices computation. The package’s architecture involves specific memory management, which lifts the limits for the data size imposed by GPU memory capacity. Additional algorithmic solutions provide a means to address the data sparsity problem and reinforce the acceleration effect for sparse datasets. Benchmarking against available central processing unit–based packages on simulated and real experimental single-cell RNA sequencing or single-cell ATAC sequencing datasets demonstrated significantly higher speed for GADES compared to other methods for both sparse and dense data processing, with additional performance boost for the sparse data.

**Conclusions:**

This work significantly contributes to the development of computational strategies for high-performance Kendall distance matrices computation and allows for the efficient processing of Big Data with the power of GPU. GADES is freely available at https://github.com/lab-medvedeva/GADES-main.

Key Points

- We present GADES, the first package for GPU-accelerated massively paralleled computation of Kendall distance matrices.
- The special memory manipulation approach allows one to bypass data size limits imposed by GPU memory capacity and to process large datasets with GPU acceleration.
- Specific algorithmic implementation gives an option to process sparse matrices (e.g., scRNA-seq or scATAC-seq data) with additional acceleration.
- GADES outperforms available packages regardless of data size, when benchmarked (in both sparse mode or dense mode) on simulated and real experimental scRNA-seq or scATAC-seq datasets.

## Introduction

Current experimental practices typically produce large arrays of multidimensional points. An extensive set of these input elements can provide meaningful information after the elements have been assigned to clusters or joined into trajectories [1, 2]. Such procedures require computation of pairwise distances between elements. Among metrics routinely used for this task is the Kendall-$\tau$ rank-based correlation coefficient [3], which is intensively applied in various fields, such as recommender systems [4, 5], bioinformatics [6, 7], and graph and network analysis [8, 9] due to the metric’s high reliability and robustness [10–12].

Large number and high dimensionality of the data points turn the calculation of the distance matrix into a massive set of simple computational tasks. The pairwise distance computation thus has high computational cost and may be the bottleneck of the data analysis [13]. At the same time, some Kendall-$\tau$ distance calculation algorithms are suitable for parallelization; in particular, the parallelization can be implemented by recruiting the graphical processing unit (GPU) instead of the central processing unit (CPU). Another important property experimental datasets often possess is a high degree of sparsity (i.e., a large number of zero elements in experimental data). This quality is notably common with regard to the single-cell data modality in bioinformatics [14]. Albeit often considered a problem [15–17], this data characteristic also may grant additional benefits for some analysis types [18], potentially including the Kendall-$\tau$ distance matrix generation. Although GPU-accelerated distance metrics computation for other metrics was recently introduced [19], no such option has been available for Kendall-$\tau$ distances yet.

In this article, we present GADES (GPU-Assisted Distance Estimation Software) that allows for the GPU-accelerated pairwise Kendall-$\tau$ distance calculation for large datasets of highly multidimensional points. GADES can be run in a parallel paradigm using the existing Compute Unified Device Architecture (CUDA) framework for GPU calculations, with a supplementary option to algorithmically take advantage of data high sparsity. We further demonstrate its application to a set of simulated and real experimental single-cell RNA sequencing (scRNA-seq) and single-cell ATAC sequencing (scATAC-seq) datasets.

### Motivation

The Kendall distance between 2 distribution vectors is calculated as follows. Given vectors $x = (x_i)_{i=1}^{F}$ and $y =(y_{i})_{i=1}^{F}$, the number of discordant pairs $D$ is calculated. The pair $(i, j), 1 < i < j \le F$ is called discordant, whether $(x_i - x_j) \cdot (y_i - y_j) < 0$. Therefore, the distance formula is $\tau (x, y) = 2 \cdot \frac{D}{F \cdot (F - 1)}$. In other words, the Kendall distance shows the normalized number of pairs, which the order in $x$ and $y$ differs.

Several CPU-based packages for calculating Kendall distances between vectors were previously introduced. They can be divided into the 2 groups: the packages of the first group compute pairwise distances of the input matrices columns and include packages in R, such as amap [20] and factoextra [21]. The second group of packages is implemented in Python (scipy [22] and pandas [23]) and only supports vector-by-vector distances calculation; input matrices, therefore, need to be split by columns to produce distance matrices (Table 1).

**Table 1:** Overview of packages applicable for Kendall distance matrices computation and their setup in benchmarking

Package	Language	Kendall	CPU mode	CPU Multicore	GPU mode	Benchmarking setup
amap	R	$\tau _{a}$	Yes	Yes	No	24 CPU threads
pandas	Python	$\tau _{a}$	Yes	No	No	1 CPU thread
scipy	Python	$\tau _{a}$	Yes	No	No	1 CPU thread
factoextra	R	$\tau _{b}$	Yes	No	No	1 CPU thread
GADES	R/CUDA	$\tau _{a}$	Yes	No	Yes	10,496 CUDA cores in GPU mode
						1 CPU thread in CPU mode

The amap package was initially designed for principal component analysis and clustering inference. Its main application is the parallel computation of distance matrices. The package factoextra provides means for the most complex set of tools for the component analysis and results visualization, but it does not support parallel computations. The scipy package allows for the basic vector and tensor manipulations with the back end enhanced with C libraries. It calculates the Kendall distance employing the Fenwick tree [24, 25], which is less computationally demanding than the pairwise inversion calculation. The pandas package allows for various statistical manipulations, including Kendall distance calculation between pairs of vectors. However, the package does not support parallel computations.

Implementing GPU-delivered massive parallelization appears to be a natural solution to accelerate Kendall distance matrix computation. In endeavoring to approach this challenge, 2 aspects need to be taken into consideration. First, GPU hardware is known to have limited RAM capacity, which reduces the amount of data that can be handled in 1 run, which implies severe restraint on the input matrix size. To bypass this restriction and to allow for large matrices to be processed, a specific memory manipulation procedure needs to be introduced to split the input data into portions (which we refer to as *batches*) that fit properly into GPU RAM. Second, GPU initialization overhead increases time of the computation process, which can have a significant effect on small dataset processing efficiency. Therefore, an option of employing CPU instead of GPU for small matrices is required.

Taking into account the high degree of the input matrix sparsity may reduce the required number of algorithm operations by omitting certain procedures with zero elements and, consequently, reinforce the boost in computational speed. Therefore, for the sparse input data, an additional processing option can be implemented (which we refer to as *sparse* mode, in contrast to *dense* mode, which makes no account for data sparsity), allowing to take advantage of this data-type specific structure.

Finally, for many commonly employed metrics, the computation procedure requires multiple matrix multiplication procedures before an aggregation step (e.g., taking the square root of the sum of 3 matrix multiplications for the Euclidean metric), which fits naturally in the most GPU-accelerated library standard methods. Kendall distance computation, however, needs the detection of the pair as a discordant step, followed by a computationally demanding aggregation step (calculating a sum of the discordant pairs detected by each pair of the features) at an earlier stage and, therefore, cannot be transduced to the matrix multiplication without a significant loss in the algorithm’s efficiency. Therefore, the proposed approach, when implemented in the GPU parallelization paradigm with no matrix multiplication, will require additional algorithmic solutions for both sparse and dense modes.

Guided by these considerations, we sought to design the GADES package, which provides calculation of Kendall-$\tau$ distance with the following features:

- an option to choose between GPU and CPU hardware for acceleration of the distance computation,
- processing input data in batches of a particular size to fit into the RAM of a given GPU setup, and
- an option to select sparse mode or dense mode for the distance matrix calculation.

## Methods

### Algorithm description

**Figure 1:**
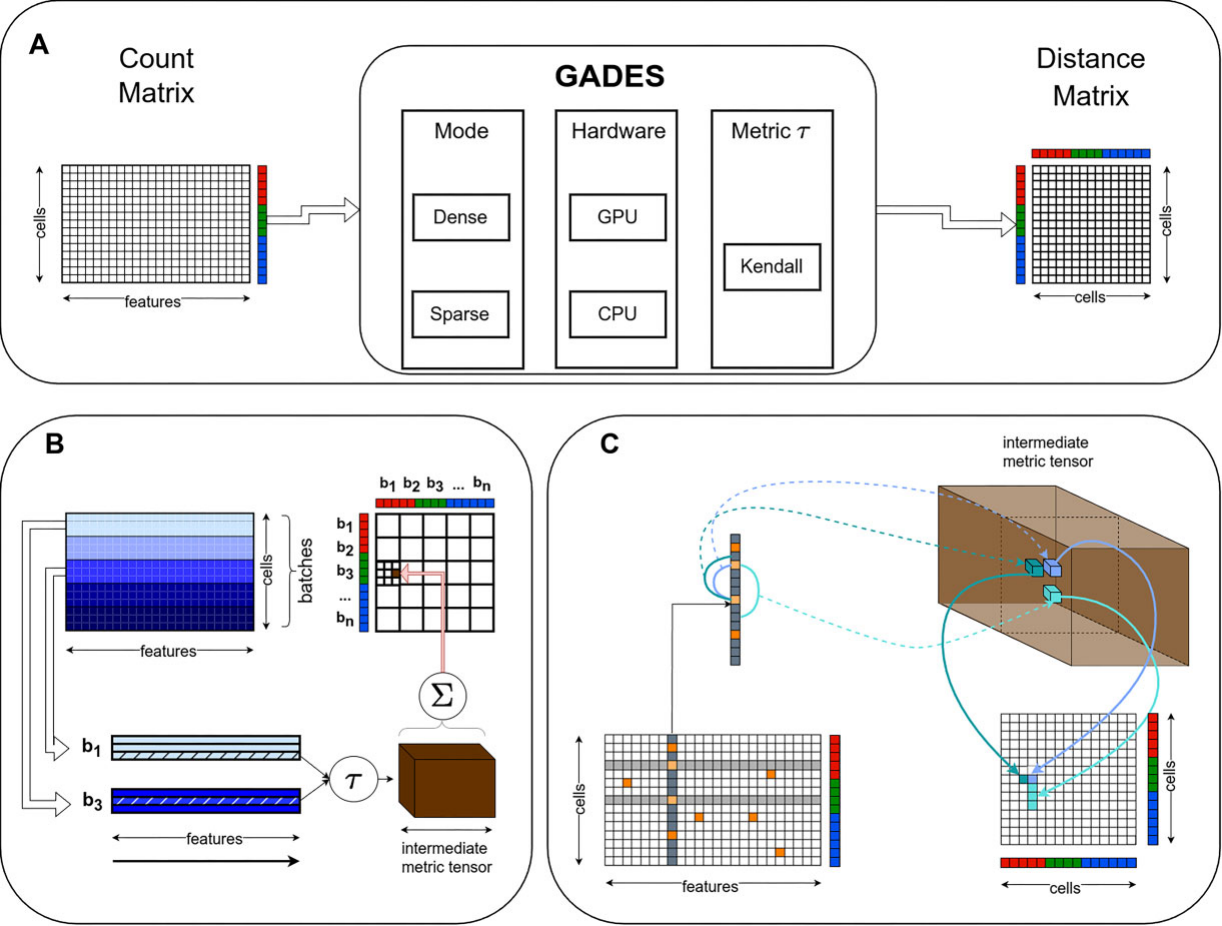
GADES pipeline overview. (A) GADES general framework. Given count matrix with $C$ cells and $F$ features, GADES calculates the Kendall distance matrix using dense or sparse mode on GPU or CPU hardware. (B) GADES batchwise count matrix processing. Count matrix $C \times F$ is split into batches of size $B \times F$ each. Distance matrices between 2 batches are calculated in parallel for the features. The results are accumulated using the atomicAdd CUDA operation. Then, block matrices of size $B \times B$ that correspond to each batch are concatenated into the final distance matrix. (C) GADES distance matrix computation with sparse layout for a specific row. One pass of the distance calculation combines the calculation of pairs of nonzero elements and sliding of one-zero range pairs.

Given matrix $M$ of size $C \times F$ (to distinguish between the 2 dimensions of $M$, we refer to them as $Features$ and $Cells$), we calculate distance matrix $DM$ of the size $C \times C$ (thus, computing distances between $cells$). Element $D_{i,j}$ is calculated using elements $DM_{i,j} = \tau (M_{i*}, M_{j*})$, where $tau$ is the Kendall correlation distance, and $M_{i*}$ denotes the corresponding row of the matrix (Fig. 1A). Since the elements are calculated independently, their calculation can be parallelized.

**Figure 2:**
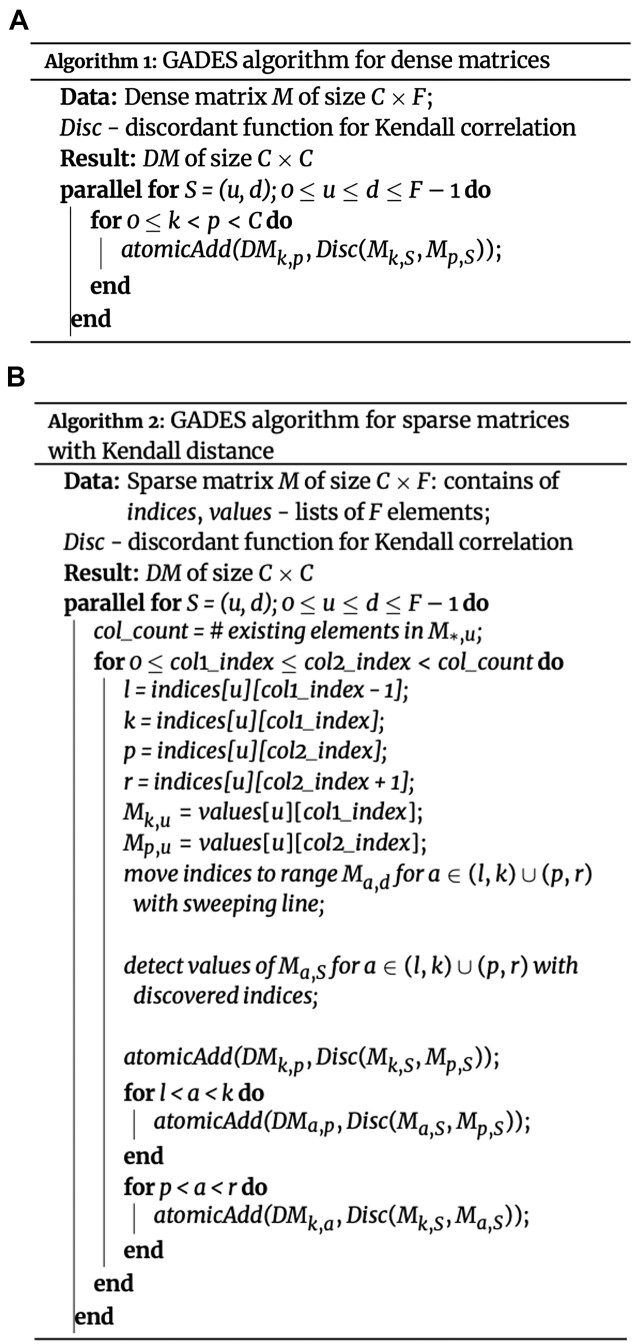
GADES algorithm pseudocode (A) for the dense mode and (B) for the sparse mode.

As distance matrix calculation requires additional memory for storing intermediate calculations, $DM$ needs to be calculated separately using batches as follows. We split matrix $M$ into $N$ matrices $\lbrace M_t\rbrace _{t=1}^{N}$ of size $B \times F$, where $B$ could be selected by the user. For each pair of batches $(m, n)$, we select rows $(k, p)$ respectively and store the values in the intermediate metric tensor structure $TM_{mB + k, nB + p}^{S}$, where $S \in [0; F - 1] \times [0; F - 1]$ for Kendall correlation. The element of distance matrix $DM_{mB + k, nB+p}$ is calculated in parallel by $k$ and $p$ using the stored $TM_{mB + k, nB + p}^{S}$ values (Fig. 2A). Since we use the atomicAdd operation, which works with the speed of the L2 cache, to aggregate values of $TM$, we do not use any shared memory or alternative caching techniques. It is quite common that the input data matrix is sparse. These sparse input matrices challenge the computation of the DM to moderate memory usage by omitting calculation for the missing elements. In addition, distance calculation should treat missing values as zeros; therefore, each element should be accounted for regarding whether at least one of $M_{k,S}$ or $M_{p,S}$ is present in the sparse matrix. We use the sweeping line method: for every pair of *nonzero* elements $M_{k, S}$, $M_{p,S}$, where $S=(u, d)$, $u \le d$, let us denote $M_{l, u}$ the first nonzero element before $M_{k, u}$ and $M_{r, u}$ the first nonzero element after the $M_{p, u}$. During calculation of $TM_{k, p}^{S}$, the method also calculates $TM_{i, p}^{S}$ for $i$ in range $(l, k)$ and $TM_{k, j}^{S}$ for $j$ in range $(p, r)$ (Fig. 1C, Fig. 2B). Thus, GADES precisely identifies all quadruples of nonzero elements $M_{i, u}$, $M_{i, d}$, $i \in (l, k)$ and $M _{j, u}$, $M_{j, d}$, $j \in (p, r)$ calculating indices of row elements that belong to corresponding intervals (Fig. 2B).

In other words, the calculation of all one-zero elements happens at the same time as their common neighboring nonzero pair, thus reducing computational time as the complexity reduces from the number of all pairs to the number of nonzero pairs. This allows GADES to increase the speed quadratically to the density of the input count matrix. Sparse matrices are loaded as the coordinate list (COO) format and converted into the compressed sparse row (CSR) format [26] to achieve a sequential scan over the indices in the sweeping line method.

### Data description

Datasets of 3 types were recruited to evaluate the packages’ performance. First, sets of simulated dense matrices of various sizes were created to explore the effects of data dimensionality. Second, simulated sets of matrices with fixed sparsity were generated to test methods on sparse data. Third, real experimental single-cell RNA-seq and ATAC-seq datasets were employed to observe the methods’ efficiency on real experimental data.

#### Simulated dense data

To explore packages’ performance, we generated 9 sets of simulated integer matrices of different sizes with a fixed random seed for experiment reproducibility. The dimensions varied from $10^2$ to $10^5$ (Supplementary Table S1). Small matrices were employed to estimate the overhead for GPU usage, whereas large matrices were used to explore the limits where CPU-based approaches could not cope with the computational task. The sparsity of data was not taken into account for these datasets, to which we refer to as *dense*.

#### Simulated sparse data

To evaluate packages’ performance on sparse data, we simulated sparse datasets by inserting a number of zero elements into previously generated dense matrices. This resulted in a set of matrices of the same size as the dense matrices and sparsity degree varying from 0.5 to 0.99 (Supplementary Table S2).

#### Experimental single-cell data

Fourteen experimental single-cell datasets were recruited to evaluate packages’ performance for real data. To take into account the dimensionality effect, we employed scRNA-seq ($N_{features} \sim \ 10^4$) and scATAC-seq ($N_{features} \sim \ 10^5$) datasets.

PBMC3K dataset [27] comprises scRNA-seq data of 2,700 peripheral blood mononuclear cells. We have extracted 3 subsets of this dataset: complete matrix (2,700 cells), B and T cells (1,806 cells), and B and CD8T cells (623 cells). The Human Lung Cell Atlas [28] contains UMI-based data of mixed blood and lung cells. We extracted 3 tissue-wise subsets: bone marrow (5,037 cells), aorta (408 cells), and lung (1,716 cells). The Fibroblasts and Cardiomyocytes Dataset [29] comprises scRNA-seq and scATAC-seq data for reprogramming cells from fibroblasts to cardiomyocytes. The scRNA-seq dataset consists of 27,999 cells and 26,124 genes, and the scATAC-seq count matrix contains 79,514 cells and 287,000 peaks. The HumanCortex dataset [30] contains gene expression programs of fetal neocortex development with 734 cells and 18,927 genes. The MouseHypothalamus dataset [31] consists of Drop-seq–based scRNA-seq of the mouse hypothalamus cell development with 14,437 cells and 23,284 features. The HSC dataset [32] contains scATAC-seq data of hematopoiesis development for 2,034 cells and 234,000 peaks. The TCells dataset [33] consists of the scATAC-seq data of T-cell proliferation with 765 cells and 49,345 peaks. The CellLines dataset [34] contains the scATAC-seq data used to detect the trans-factors of cell-to-cell interactions with 1,224 cells and 125,648 peaks. The summary for the datasets is presented in Supplementary Table S3.

### Evaluation of performance

To evaluate GADES’ performance, we tested it against available non-GPU packages for Kendall distance computation: factoextra [21], amap [20], scipy 1.10.1 [22], and pandas 1.5.3 [23]. Package amap was run with CPU acceleration, and packages factoextra, pandas and scipy were run without hardware acceleration.

On the dense simulated datasets, GADES was run in 2 setups: one with GPU acceleration in dense mode and the second with CPU acceleration in dense mode (which we refer to as *GADES-GPU-dense* and *GADES-CPU-dense*, respectively). On the sparse simulated and real experimental datasets, both the hardware acceleration type (GPU/CPU modes) and the accounting for data sparsity (sparse/dense modes) were varied, thus resulting in 4 GADES setups (*GADES-GPU-dense, GADES-CPU-dense, GADES-GPU-sparse*, and *GADES-CPU-sparse*).

All the packages were deployed at the HPC cluster with NVIDIA RTX 3090 with 24 GB VRAM and 24 CPUs available. We used cores without hyperthreading or virtualization mechanisms enabled. All the experiments were run with the same setup: 1 GPU for GADES and 24 CPUs for multiprocessing packages (amap); if the package did not support multithreading, we used 1 CPU (Table 1). To ensure the amap method employed physically all the allocated cores (24, see above) rather than approximately half that would be expected for a virtual parallelization setup, we measured the average CPU usage for the 24 threads and observed the CPU core usage being at the level of 2,200% of a single CPU performance. Since Kendall distance computation, differing from other metrics, cannot be reduced to matrix multiplication, implicit built-in parallelization in some libraries was not set, and the number of processes for the packages was specified explicitly.

We applied all the packages to a number of simulated and real single-cell datasets (see Data Description for details). Each matrix was processed with each method 25 times to take processor throttling into account; every real dataset was processed with every method 10 times. Time limit for every run of a method was set up at 1 day (86,400 seconds). For all the experiments, computational time $T_{package}$ and relative *acceleration* as a scaling factor $A = \frac{T_{package}}{T_{GADES-GPU-dense}}$ of a package were compared to GADES-GPU-dense running time. The results visualization was plotted with the seaborn package [35].

### Evaluation of optimal batch size

To assess the effect of the batch size on the performance of the GADES-GPU method, we applied the following procedure. All the analyzed datasets were additionally processed with the GADES-GPU-dense method for generated dense datasets and with the GADES-GPU-sparse method for generated sparse and experimental single-cell datasets. For generated datasets, we selected batch sizes being divisors of the cells number, namely, 100, 500, 1,000, 5,000, and 10,000 cells per batch. For the experimental single cells, we selected batch sizes of 250, 500, 1,000, 2,000, and 4,000 cells.

As the optimal performance was achieved at a batch size of 500 cells (see Results), we represented the performance for each analyzed batch size $B$ as the performance factor $A_B = \frac{T_{500}}{T_{B}}$, where $T_{500}$ is the computational time for a batch size of 500 cells.

### Evaluation of the memory usage

To explore the RAM usage of all the tested packages, including GADES, we employed memory profiling tools: profmem package [36] for the R language and with psutil package [37] for the Python language. We measured the peak memory usage by disabling the implicit calls of the garbage collector. To avoid extra calculations, we subtracted the allocated memory size, which is required for storing the obtained distance matrix, from the result. For the GADES method, we tested all the modes with batch size 500, which was shown to be the optimal batch size for the computational time.

## Results

To evaluate the performance of the packages, we computed their relative acceleration compared to GADES-GPU-dense running time $A = \frac{T_{package}}{T_{GADES}}$, thus using GADES-GPU-dense as the baseline (see Methods: Evaluation of performance). $\log A < 0$ implies the package runs more slowly than GADES-GPU-dense on the same data, and $\log A > 0$ means the package demonstrates higher efficiency compared to GADES-GPU-dense. For cases when the time limit was reached and the computation failed, the results computation time was set to the value of a time limit of 86,400 s.

### Case study: simulated dense data

We explored the patterns of acceleration for the 3 sets of dense matrices depending on their size $|W| = N_{features} \times N_{cells}$ ($|W| \in \lbrace 10^5, 10^6 , 10^7\rbrace$) (see Methods: Data description).

**Figure 3:**
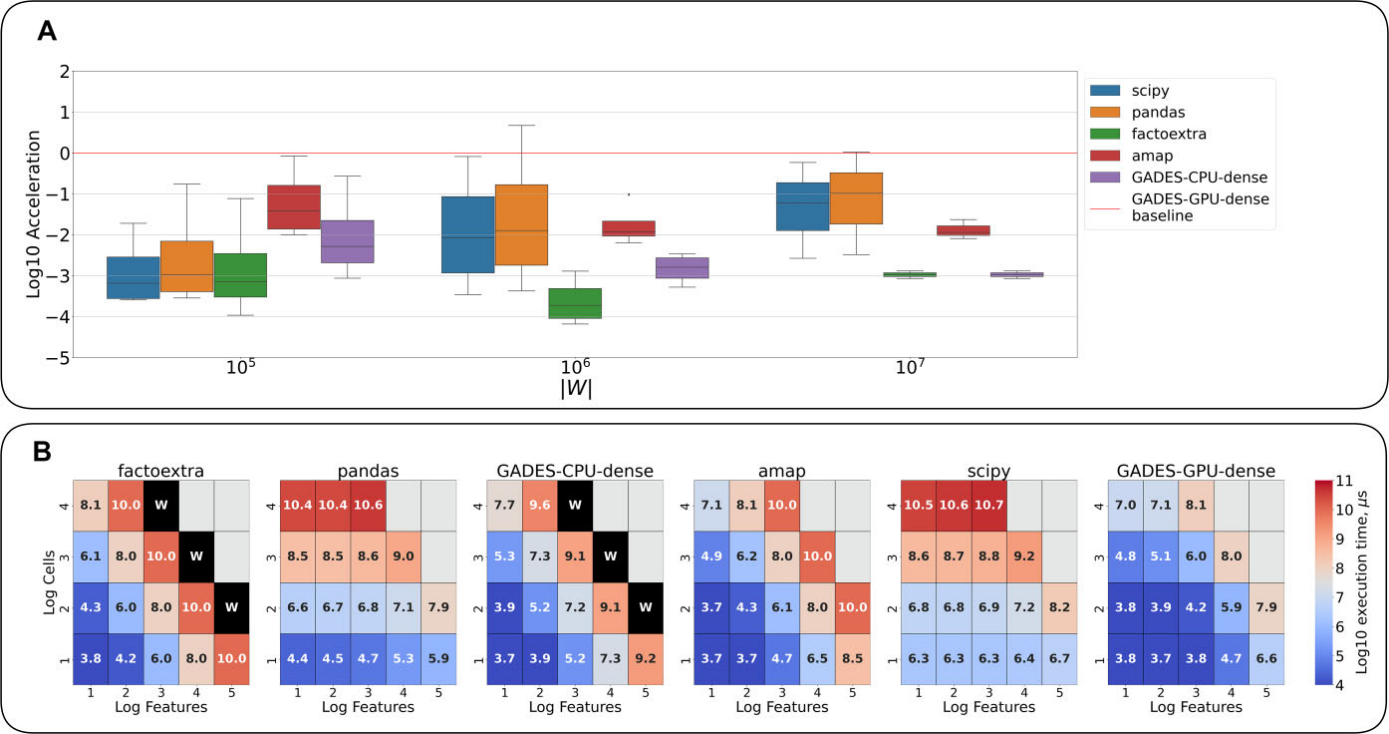
Benchmarking results for dense generated datasets and all the benchmarked packages. (A) Log-scaled packages’ acceleration for different input matrix size |W| in comparison with GADES-GPU-dense processing time as the baseline. (B) Heatmaps of the log-scaled mean computation time for every set of dimensions (cells and features); results for matrices failed to process in 24 hours walltime, marked W.

GADES-GPU-dense demonstrated significantly higher data processing speed than the rest of the packages and all $|W|$ values (Fig. 3A, Supplementary Fig. S1). The worst performance was shown by factoextra and GADES-CPU-dense, which ran 2,800 and 340 times more slowly than GADES-GPU-dense. The efficiency of scipy and pandas was higher, and their speed was 120 and 75 times lower than the speed of GADES-GPU-dense. These packages’ performance also moderately improved with the increase of $|W|$ due to their asymptotic complexity [22, 23].

Some boxplots displayed a significant level of variance across processing time for matrices with the same $|W|$, delivered by different combinations of dimensions $N_{cells}$ and $N_{features}$, which may imply an unequal impact of dimensions’ ($N_{cells}$ and $N_{features}$) increase on the running speed. To examine the magnitude of this effect, we analyzed separately the running time observed for each ($N_{cells}$, $N_{features}$) pair in the processed datasets (Fig. 3B). For GADES-GPU-dense, scipy and pandas packages’ increase in $N_{cells}$ affected the running time more intensely than in $N_{features}$. Differently, for the amap package, the impact of $N_{features}$ increment was stronger than the one of $N_{cells}$. For GADES-CPU-dense and factoextra, the influence of $N_{cells}$ and $N_{features}$ augmentation proved to be comparable.

### Case study: simulated sparse data

**Figure 4:**
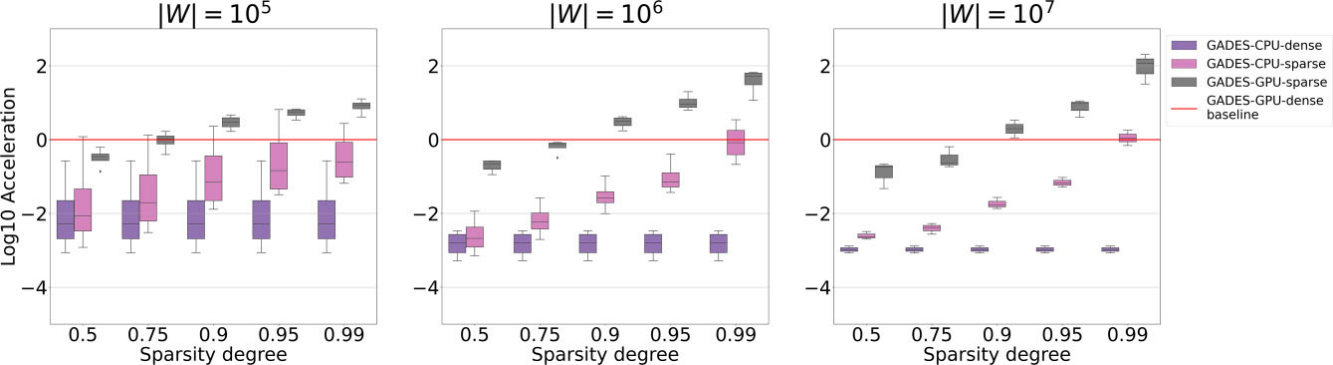
Benchmarking results for sparse generated datasets and all GADES modes: the impact of the data sparsity degree on log-scaled acceleration in comparison with GADES-GPU-dense processing time as the baseline.

For the simulated datasets of varied sparsity degree (see Methods: simulated sparse data for details), we have observed no effect of sparsity on the performance of existing CPU-based packages, as well as on GADES-CPU-dense and GADES-GPU-dense (Fig. 4, Supplementary Fig. S2). GADES-CPU-sparse and GADES-GPU-sparse, in turn, demonstrated a significant rise in running speed with the increase of input data sparsity. The magnitude of the effect augmented with the size of the input data $|W|$ increment. Thus, GADES-CPU-sparse ran 6.8 times more quickly than the GADES-CPU-dense baseline for $|W| = 10^5$, 14 times more quickly for $|W| = 10^6$, and 16 times more quickly for $|W| = 10^7$. For the degree of sparsity 0.99, GADES-CPU-sparse performance became comparable to the one of GPU-dense, as GADES-CPU-sparse ran 520 times more quickly than GADES-CPU-dense for $|W| = 10^6$ and 850 times more quickly for $|W| = 10^7$.

### Case study: experimental single-cell datasets

**Figure 5:**
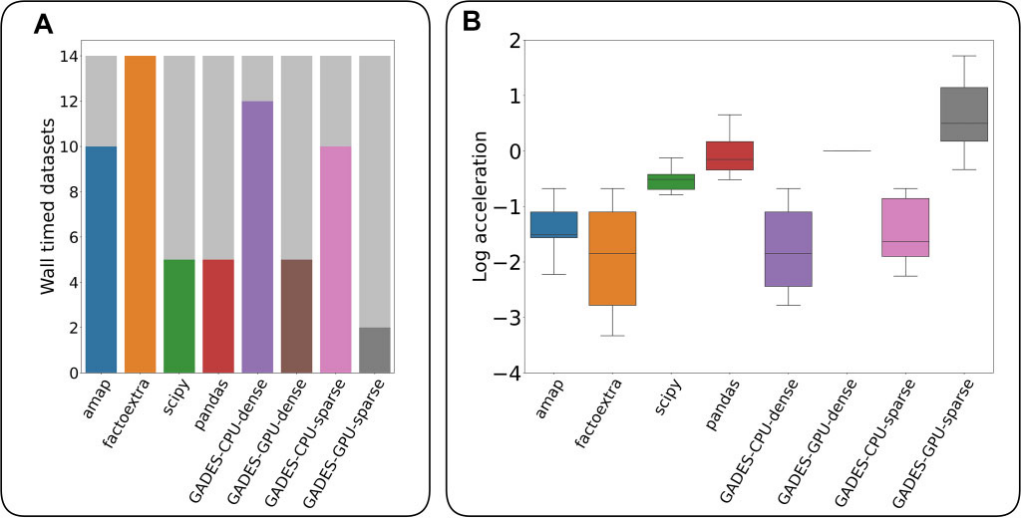
Benchmarking results for the real experimental datasets. (A) Number of datasets failed to process within the running time limit we set for all the packages. (B) Log-scaled acceleration for all the packages in comparison with GADES-GPU-dense processing time as the baseline.

Run on real single-cell datasets, packages demonstrated a high failure rate (i.e., number of datasets failed to be processed within the time limit) (Fig. 5A). Four datasets (PBMC5K, MouseHypothalamus, Fibrocard-RNA, Fibrocard-ATAC) out of 14 could not be processed within the time limit by any of the packages. The only exception was GADES-GPU-sparse, which managed to process 2 of them (MouseHypothalamus, Fibrocard-RNA) and had the lowest failure rate across all the packages. The 2 datasets that all the packages failed to process were excluded from the further analysis.

For the remaining properly processed datasets, GADES-GPU-sparse proved to be the most efficient approach (Fig. 5B). Its running speed was 7 times higher than the GADES-GPU-dense baseline. The pandas package showed a reasonable performance comparable to the baseline (although 12% lower), yet ran 7.8 times more slowly than GADES-GPU-sparse. All other packages demonstrated lower speed than baseline (from 21 times more slowly for the factoextra package to a 2.7 times decrease in speed for scipy). Thus, GADES-GPU-sparse displayed the best efficiency in terms of number of processed (i.e., not failed due to the time limit) datasets and the speed of their processing.

### Computational cost Study: GPU efficacy dependence on the batch size

**Figure 6:**
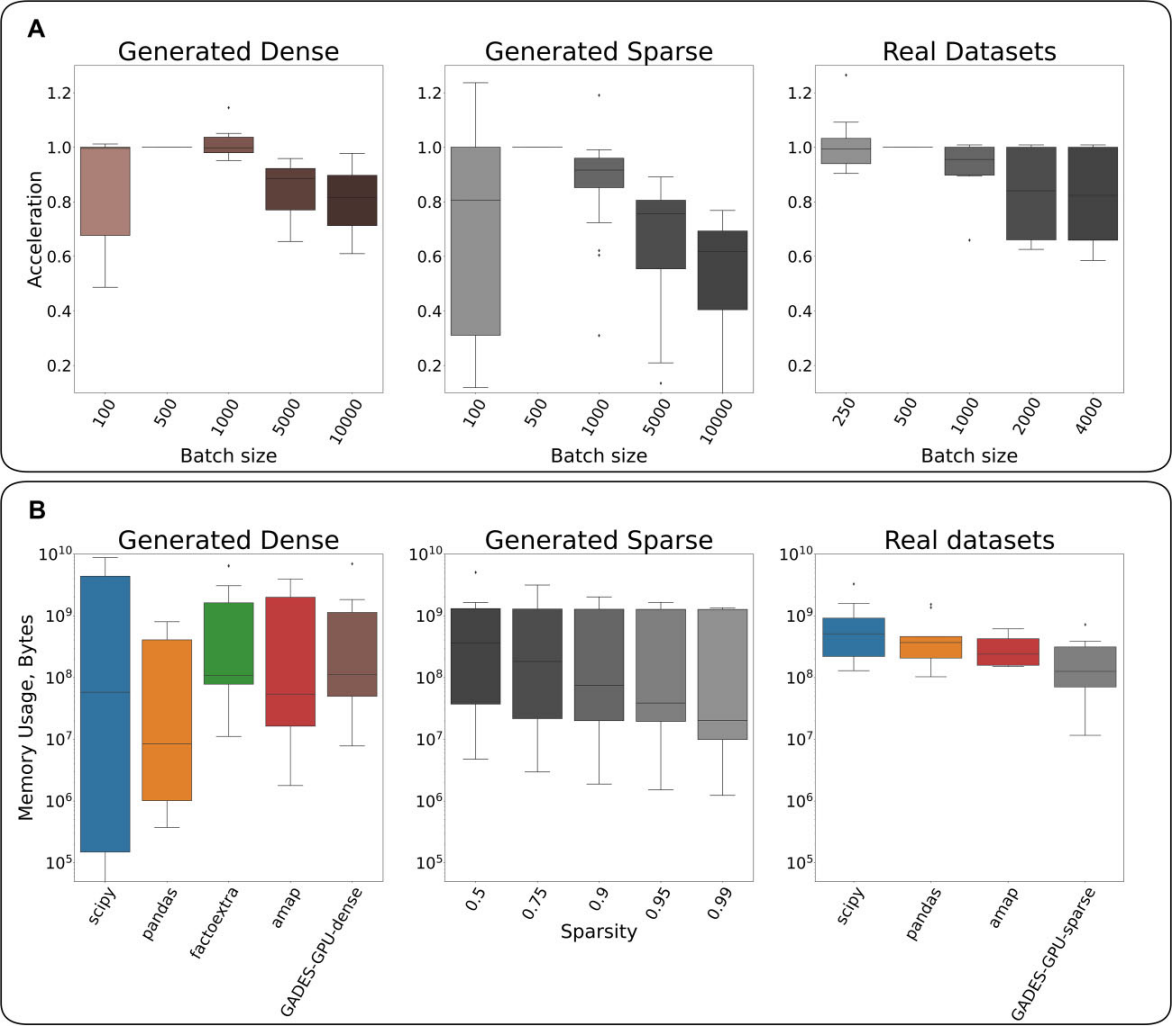
Memory usage and the effects of batch size. (A) GADES performance (in dense mode for generated dense datasets and in sparse mode for generated sparse and real datasets) for all the datasets and various batch sizes with baseline of the batch size of 500 elements. (B) Memory usage of all the tested methods for the generated dense and real datasets and GADES-sparse for the generated sparse datasets.

For the real and generated datasets, package GADES-GPU demonstrated the same pattern of the efficiency dependence on the batch size (Fig. 6). For the generated sparse and real datasets, the optimal batch size is typically 500 cells. For the real datasets, the GPU efficacy performance factor (see Methods) for the batch size of 250 can be more than 1 or less than 1, and it depends on the input matrix, whereas for the generated sparse dataset, the lower batch size is robustly more than 1. At the same time, for the generated sparse datasets, the factor for the batch size of 1,000 cells could be more or less than 1 (Fig. 6A, Supplementary Fig. S3). Therefore, the most stable results are observed for a batch size of 500, which is recommended for the default parameter for analysis, as it can deliver a reasonable trade-off between the cost of context switching for lower batch sizes and computationally challenging interactions while calculating the distance matrix for the same batch.

### Computational cost Study: Memory usage

With respect to the memory usage, we observed differing patterns for Python-based and R-based packages, as Kendall correlation for the scipy and pandas packages was calculated in place. Among the R packages, the GADES-GPU-dense package had the lowest memory usage (Fig. 6B). Furthermore, an increase in the degree of sparsity for the generated sparse datasets was associated with a redaction in memory usage for by GADES-GPU-sparse method, and the memory usage heavily depended on the nonzero element count. To affirm the observation, we measured RAM usage of GADES-GPU-sparse for all real datasets using a batch size of 500 elements. We fitted model $\log (Memory) \sim \log (NonZeroElements)$. Surprisingly, we observed the explained variability $R^2 = 0.85$, while for other benchmarked packages, the values were lower (0.72, $-0.1$, and 0.73 for scipy, amap, and pandas packages correspondingly) (Supplementary Fig. S4, Supplementary Table S5). Thus, regarding the memory usage, the GADES-GPU package is comparable to CPU-based packages for the Kendall distance matrix calculation in dense mode and has a memory advantage in sparse mode.

## Discussion

In this study, we have presented GADES, the first computational package to compute Kendall-$\tau$ distance matrices using the advantage of the massively parallel computation delivered by the GPU acceleration. The implemented specific memory management lifts the limits for the data size imposed by GPU memory capacity, allowing processing datasets of any size. In addition, the algorithmic realization of the package’s architecture allows one to take advantage of the input data sparsity, providing an additional boost to the computational performance. GADES can be run on either CPU or GPU hardware, which makes it flexible for various computational architecture setups and allows for optimal processing of datasets of different sizes.

For the simulated dense datasets, we have observed GADES in GPU and dense mode to drastically outperform all other packages regardless of the dataset size and running 75 times more quickly than pandas, its nearest rival. For the simulated datasets with a high degree of sparsity (more than 75%–90% zeros in the dataset), GADES in GPU and sparse-aware mode outperforms other packages acutely, regardless of the dataset size, with a tendency to perform better for the larger size of the input data or a higher degree of sparsity. However, the effect observed for the real scATAC-seq and scRNA-seq datasets is less radical, although GADES with GPU acceleration in sparse-aware mode still performs computations significantly more quickly than the rest of the packages (7.8 times more quickly than the second best package). This displays the effect that the structure of input data, including nonrandom distribution of zero elements in the input matrix, may have. In terms of RAM resources efficiency, Python-based packages showed a better performance compared to R-based packages for dense data, which is not surprising since Python packages handle float arrays, whereas R packages work with double data type; additionally, Python-based packages perform operations in place. GADES-dense mode proved to be comparable to other R packages in RAM resources efficiency and showed a significant boost in performance for sparse mode.

Kendall-$\tau$ correlation, being a robust nonparametric similarity measure, is widely employed for multidimensional data analysis in cases when the normality propositions are weakly based. However, its applicability is often limited, especially in the Big Data domain, by the high computational costs and unreasonable running time. The GADES package, the first method with GPU acceleration and batched memory manipulation and algorithmically boosted for highly sparse data, lifts these constraints. This makes GADES a powerful tool, providing a way to broadly and efficiently apply the Kendall-$\tau$ correlation to large datasets in numerous engineering and scientific fields.

The current realization of GADES has several limitations. First, when GADES is in GPU mode, data transfer needs to be done into GPU RAM, which CPU-based packages are naturally free from. This may have a negative impact on the package’s running time for the datasets of smaller size, but it can smoothly be addressed by falling to CPU mode of GADES. Second, GADES-GPU may demand more RAM resources for data processing compared to CPU-based packages in some scenarios. In addition, the current implementation of GADES lacks an option for parallelization in CPU mode, which for some benchmarking setups allowed other CPU-based packages to be more efficient than GADES-CPU.

One of the main directions for extending GADES is altering the method’s architecture to introduce CPU parallelization, which will improve the efficiency of low-dimensionality data processing. Incorporating procedures to calculate additional distance types, such as Euclidean, Spearman, or cosine distances, is one more avenue to improve the functionalities of the package. Another task to address is enhancing the versatility of the GADES approach via transferring the designed computational core to alternative platforms, such as introducing the Python version of the GADES package.

The presented package, GADES, allows for more efficient large-dimension or sparse data preprocessing, including, but not limited to, the results of the single-cell next-generatioon sequencing experiments (e.g., scRNA-seq and scATAC-seq). The package provides the means to improve efficiency, sensitivity, and running speed of the existing analysis practice in bioinformatics and other areas.

## Availability of Source Code and Requirements

**GADES source code**

**Project name:** GADES-main

**License:** GPL-3.0 license

**Project homepage:** https://github.com/lab-medvedeva/GADES-main

**PID:** swh:1:snp:8550b8daa79a0ca2b0ce11dba2c6e9f880fb4009

**Citation:** Akhtyamov P, Nabi A, Gafurov V, Sizykh A, Favorov A, Medvedeva Y, Stupnikov A. GADES—GPU-assisted distance estimation software [Computer software]. Software Heritage, https://archive.softwareheritage.org/swh:1:snp:8550b8daa79a0ca2b0ce11dba2c6e9f880fb4009;origin=https://github.com/lab-medvedeva/GADES-main

**Operating system(s):** Platform independent

**Programming language:** R 4.1+, CUDA 11+

**Other requirements:** see public environment file

**RRID:** SCR_02551

**GADES reproducibility code**

**Project name:** Article-GADES

**License:** GPL-3.0 license

**Project homepage:** https://github.com/lab-medvedeva/Article-GADES

**PID:** swh:1:snp:48fb0f5536ca7ee4cd53156e0b5e7373f2c4af4e

**Citation:** Akhtyamov P, Nabi A, Gafurov V, Sizykh A, Favorov A, Medvedeva Y, Stupnikov A. Article-GADES [Computer software]. Software Heritage, https://archive.softwareheritage.org/swh:1:snp:48fb0f5536ca7ee4cd53156e0b5e7373f2c4af4e;origin=https://github.com/lab-medvedeva/Article-GADES

**Operating system(s):** Platform independent

**Programming language:** bash, R 4.1+, Python 3.7+

**Other requirements:** see public environment file

**GADES reproducibility workflow**

**License:** GPL-v3

**Link:** https://doi.org/10.48546/WORKFLOWHUB.WORKFLOW.1125.1

**PID:** 10.48546/WORKFLOWHUB.WORKFLOW.1125.1

**Citation:** Akhtyamov P. GADES reproducibility workflow. WorkflowHub. https://doi.org/10.48546/WORKFLOWHUB.WORKFLOW.1125.1

## Additional Files

**Supplementary Fig. S1**. Benchmarking results for dense generated datasets and all the benchmarked packages. Log-scaled packages running time for different input matrix size $|W|$.

**Supplementary Fig. S2**. Benchmarking results for sparse generated datasets and all GADES modes: the impact of the data sparsity degree on log-scaled packages’ running time.

**Supplementary Fig. S3**. Benchmarking results for dense generated datasets for batch size usage. Acceleration running time for different input matrix size $|W|$.

**Supplementary Fig. S4**. Lineplots of the memory usage for the tested methods to the number of nonzero elements and the $|W|$. GADES-GPU-sparse memory usage is proportional to the number of nonzero elements, whereas the other methods are proportional to the number of elements.

**Supplementary Table S1**. Summary for simulated matrices used for benchmarking packages on dense data.

**Supplementary Table S2**. Summary for simulated matrices used for benchmarking packages on sparse data.

**Supplementary Table S3**. Summary for the real experimental datasets used for benchmarking.

**Supplementary Table S4**. Benchmarking results for dense generated datasets.

**Supplementary Table S5**. Adjusted $R^2$ values for linear models of logarithmic memory usage to $\log |W|$ and logarithmic nonzero elements, respectively.

giae088_Supplementary_File

giae088_GIGA-D-24-00103_Original_Submission

giae088_GIGA-D-24-00103_Revisi3n_1

giae088_GIGA-D-24-00103_Revision_1

giae088_GIGA-D-24-00103_Revision_2

giae088_Response_to_Reviewer_Comments_Original_Submission

giae088_Response_to_Reviewer_Comments_Revision_1

giae088_Response_to_Reviewer_Comments_Revision_2

giae088_Reviewer_1_Report_Original_Submission Zexuan Zhu -- 5/7/2024

giae088_Reviewer_1_Report_Revision_1 Zexuan Zhu -- 9/13/2024

giae088_Reviewer_2_Report_Original_Submission Huang Zhi-An -- 6/11/2024

giae088_Reviewer_2_Report_Revision_1 Huang Zhi-An -- 9/12/2024

## Abbreviations

COO: coordinate list format; CPU: central processing unit; CSR: compressed sparse row format; CUDA: Compute Unified Device Architecture; GADES: GPU-Assisted Distance Matrix Estimation Software; GADES-CPU-dense: GADES package run with CPU acceleration and no account for data sparsity; GADES-CPU-sparse: GADES package run with CPU acceleration and specific mode accounting for data sparsity; GADES-GPU-dense: GADES package run with GPU acceleration and no account for data sparsity; GADES-GPU-sparse: GADES package run with GPU acceleration and specific mode accounting for data sparsity; GPU: graphical processing unit; RAM: random access memory; scATAC-seq: single-cell ATAC sequencing; scRNA-seq: single-cell RNA sequencing.

## Potential Implications

GADES can be effective in statistical inference of large or sparse datasets, such as single-cell next-generation sequencing datasets, social graph networks, and stiffness matrices, and can be adapted for various types of analysis in bioinformatics, engineering, machine learning, and other fields.

## Data Availability

Snapshots of the code and workflows are available in SoftwareHeritage [38, 39] and Workflowhub.eu [40]. **Simulated data** All the code for data download or simulation, extraction, and preprocessing can be found at Article-GADES: https://github.com/lab-medvedeva/Article-GADES.. **Public data** Publicly available datasets were analyzed in this study: PBMC3K: SRP073767 PBMC3K B& T: SRP073767 PBMC3K: B& CD8T: SRP073767 HLCA marrow: GSE109774 HLCA aorta: GSE109774 HLCA lung: GSE109774 HSC: GSE96769 HumanCortex: GSE75140 FibroCard ATAC: GSE165837 FibroCard RNA: GSE165838 MouseHypothalamus: GSE87544 PBMC5K: GSE129785 TCells: GSE107223 CellLines: GSE65360
