# Supplementary material for: GPU-accelerated Kendall distance computation for large or sparse data
Source: Gigascience. 2024 Dec 9;13:giae088. doi: 10.1093/gigascience/giae088 (PMC11631066; doi:10.1093/gigascience/giae088)
Supplement: giae088_Supplementary_File [file giae088_supplementary_file.pdf]

Supplementary materials

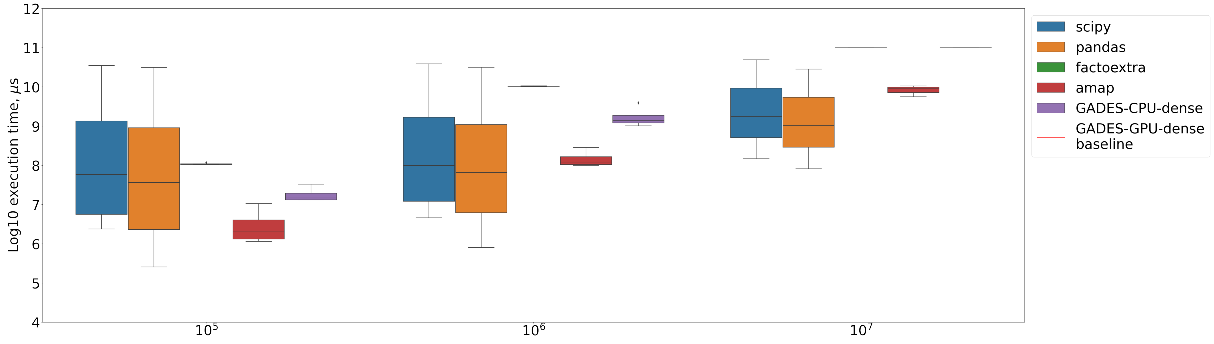

Supplementary Figure 1. Benchmarking results for dense generated datasets and all the benchmarked packages. Log-scaled packages running time for different input matrix size $|W|$.

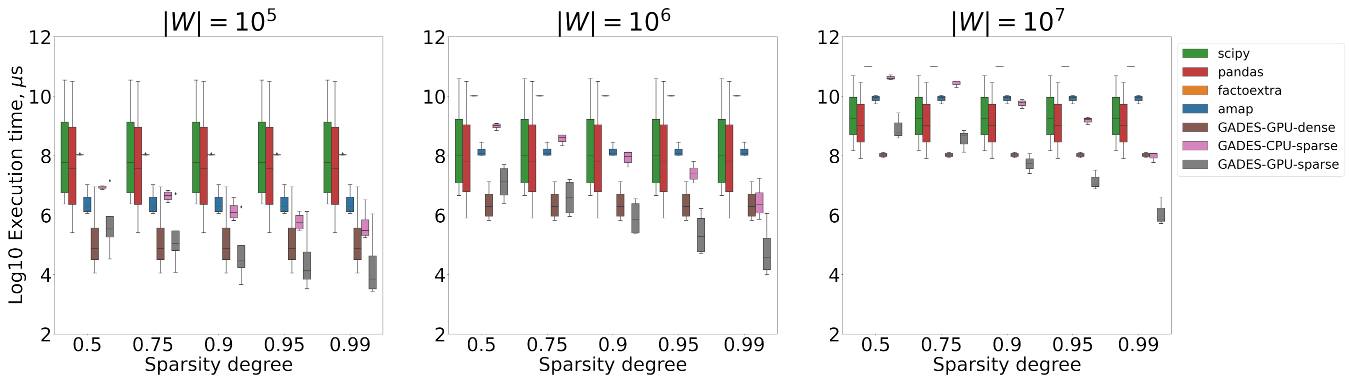

Supplementary Figure 2. Benchmarking results for sparse generated datasets and all GADES modes: the impact of the data sparsity degree on Log-scaled packages running time

Supplementary Table 1. Summary for simulated matrices used for benchmarking packages on dense data

Number of cells	Number of features	Product ($\|W\|$)	Number of iterations
10	10	100	25
10	100	1000	25
10	1000	10000	25
10	10000	100000	25
10	100000	1000000	25
100	10	1000	25
100	100	10000	25
100	1000	100000	25
100	10000	1000000	25
100	100000	10000000	25
1000	10	10000	25
1000	100	100000	25
1000	1000	1000000	25
1000	10000	10000000	25
10000	10	100000	25
10000	100	1000000	25
10000	1000	10000000	25

Supplementary Table 2. Summary for simulated matrices used for benchmarking packages on sparse data

Number of cells	Number of features	Product ($\|W\|$)	Number of iterations	Sparsity degree
10	10000	100000	25	0.5;0.75;0.9;0.95;0.99
10	100000	1000000	25	0.5;0.75;0.9;0.95;0.99
100	1000	100000	25	0.5;0.75;0.9;0.95;0.99
100	10000	1000000	25	0.5;0.75;0.9;0.95;0.99
100	100000	10000000	25	0.5;0.75;0.9;0.95;0.99
1000	100	100000	25	0.5;0.75;0.9;0.95;0.99
1000	1000	1000000	25	0.5;0.75;0.9;0.95;0.99
1000	10000	10000000	25	0.5;0.75;0.9;0.95;0.99
10000	10	100000	25	0.5;0.75;0.9;0.95;0.99
10000	100	1000000	25	0.5;0.75;0.9;0.95;0.99
10000	1000	10000000	25	0.5;0.75;0.9;0.95;0.99

Supplementary Table 3. Summary for the real experimental datasets used for benchmarking

Dataset ID	Data type	Number of cells	Number of features	Sparsity	GSE/SRA	Description
PBMC3K	scRNA-seq	2700	13714	0.938	SRP073767	3000 cells as example of 10X Genomics tools
PBMC3K BT	scRNA-seq	1806	13714	0,942	SRP073767	Selected B cells and T cells
PBMC3K BCD8T	scRNA-seq	623	13714	0,944	SRP073767	Selected B cells and CD8 T cells
HLCA: marrow	scRNA-seq	5037	23341	0,918	GSE109774	Marrow cells
HLCA: aorta	scRNA-seq	408	23341	0,892	GSE109774	Aorta cells
HLCA: lung	scRNA-seq	1716	23341	0,892	GSE109774	Lung cells
HSC	scATAC-seq	2034	234000	0,975	GSE96769	scATAC-seq benchmark dataset
HumanCortex	scRNA-seq	734	18927	0,801	GSE75140	Dataset with low sparsity
FibroCard	scATAC-seq	79514	287000	0,993	GSE165837	Reprogramming Fibroblasts to Cardiomyocytes
FibroCard	scRNA-seq	27999	26124	0,95	GSE165838	Reprogramming Fibroblasts: big number of cells
MouseHypothalamus	scRNA-seq	4029	20058	0,934	GSE87544	United dataset of Mouse and Human
PBMC5K	scATAC-seq	10032	106935	0,933	GSE129785	Integrated dataset of 5000 human and 5000 mouse cells
TCells	scATAC-seq	765	49345	0,966	GSE107223	Four sub types of T Cells
CellLines	scATAC-seq	1224	125648	0,964	GSE65360	Single cell epigenomes with 8 cell types and 4 targeted cell manipulations

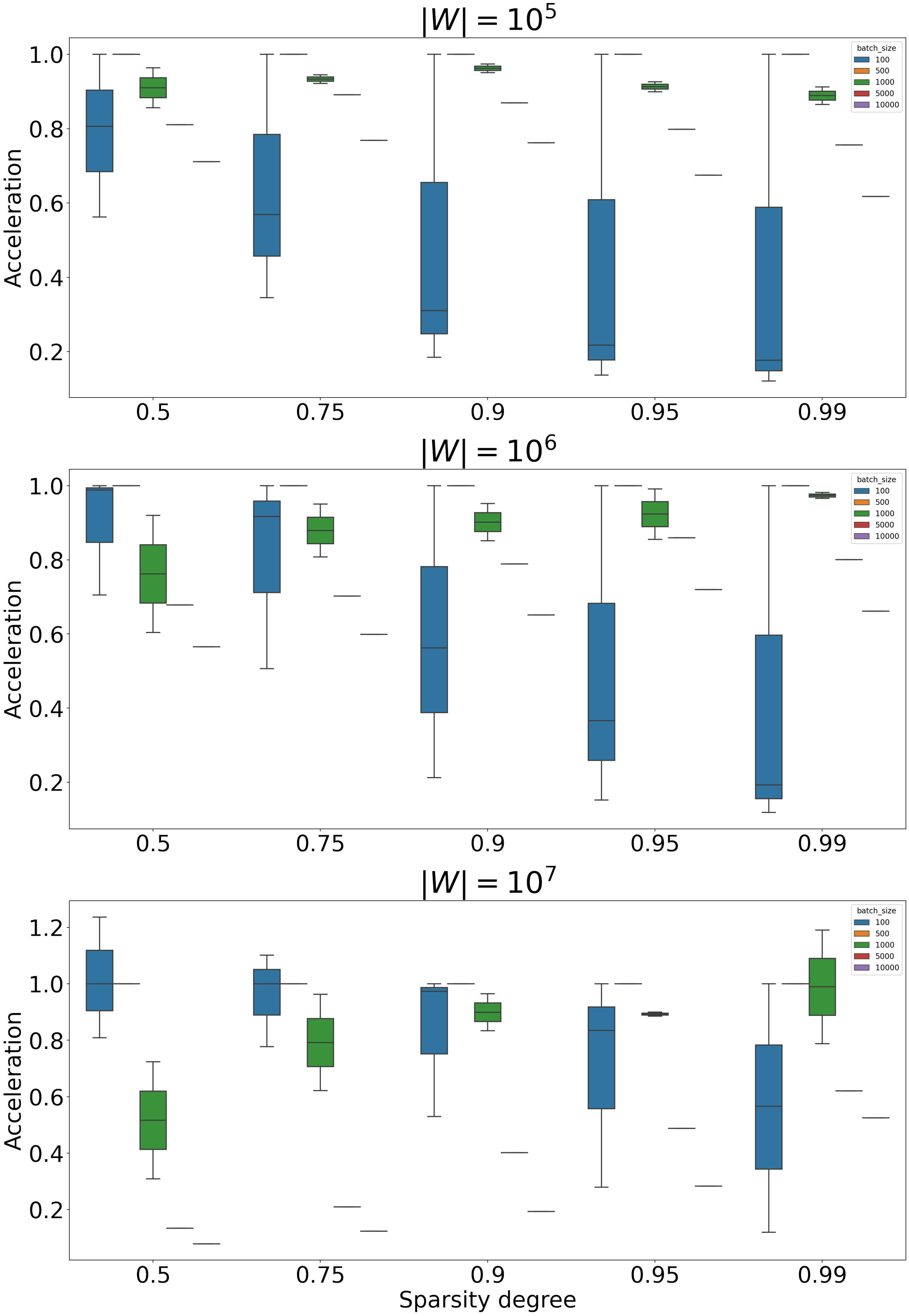

Supplementary Figure 3. Benchmarking results for dense generated datasets for batch size usage. Acceleration running time for different input matrix size $|W|$.

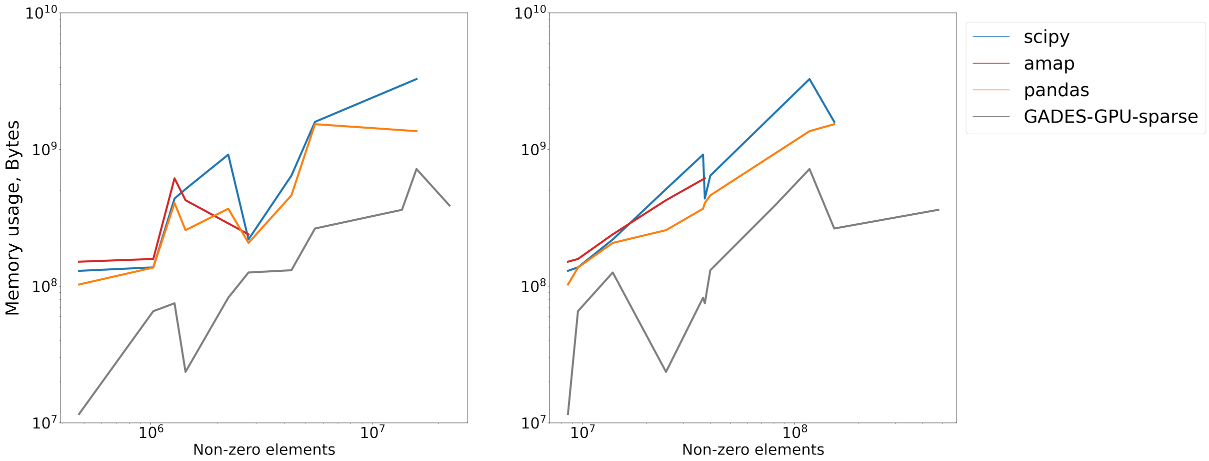

Supplementary Figure 4. Lineplots of the memory usage for the tested methods to the number of non-zero elements and the $|W|$. GADES-GPU-sparse memory usage is proportional to the number of non-zero elements, whereas the other methods are proportional to the number of elements.

Supplementary Table 4. Benchmarking results for dense generated datasets

method	cells	features	|W|	average	logMeanTime
GADES-CPU-dense	10	10	100	2312.78	3.3641343230731886
GADES-GPU-dense	10	10	100	2395.925	3.379473219144677
amap	10	10	100	2442.19	3.3877794486103574
factoextra	10	10	100	2205.7	3.3435464431882567
pandas	10	10	100	49655.12752532959	4.695964101592264
pythonic	10	10	100	88454.65183258057	4.946720677405116
GADES-CPU-dense	10	100	1000	4489.67	3.652214420636741
GADES-GPU-dense	10	100	1000	2194.0	3.3412366232386925
amap	10	100	1000	2510.2	3.3997083252404208
factoextra	10	100	1000	13157.4	4.119170077939687
pandas	10	100	1000	30559.01527404785	4.485139355533968
pythonic	10	100	1000	89084.17224884033	4.9498005489796
GADES-CPU-dense	100	10	1000	4495.33	3.6527615785872634
GADES-GPU-dense	100	10	1000	2818.275	3.449983368175556
amap	100	10	1000	2872.95	3.458328067720784
factoextra	100	10	1000	16833.65	4.226178293218348
pandas	100	10	1000	3817218.470573425	6.581747016603356
pythonic	100	10	1000	273004.6272277832	5.4361700080754165
GADES-CPU-dense	10	1000	10000	210507.01	5.323266562659228
GADES-GPU-dense	10	1000	10000	2682.0	3.42845877351558
amap	10	1000	10000	35161.31	4.546065047151819
factoextra	10	1000	10000	1084186.26	6.035103899122409
pandas	10	1000	10000	76478.36208343506	4.883538578199226
pythonic	10	1000	10000	96476.10187530518	4.984419747446927
GADES-CPU-dense	100	100	10000	172509.51	5.236813041592939
GADES-GPU-dense	100	100	10000	3594.875	3.5556837938137416
amap	100	100	10000	18282.15	4.262027267887415
factoextra	100	100	10000	1062423.45	6.026297647955047
pandas	100	100	10000	4203282.4993133545	6.623588579577162
pythonic	100	100	10000	280936.52725219727	5.448608209663434
GADES-CPU-dense	1000	10	10000	174562.98	5.241952147236644
GADES-GPU-dense	1000	10	10000	63817.675	4.804940977966807
amap	1000	10	10000	70160.51	4.846092737276182
factoextra	1000	10	10000	1174869.0	6.069989444693863
pandas	1000	10	10000	282040597.486496	8.450311626033416
pythonic	1000	10	10000	18244537.4250412	7.261132856667573
GADES-CPU-dense	10	10000	100000	16353412.18	7.213608383071803
GADES-GPU-dense	10	10000	100000	45165.975	4.654811389804784
amap	10	10000	100000	2931787.74	6.467132524398946
factoextra	10	10000	100000	106958798.6	8.029216516128265
pandas	10	10000	100000	258089.85233306885	5.411770929137473
pythonic	10	10000	100000	99220.75271606444	4.996602517390685
GADES-CPU-dense	100	1000	100000	13162224.78	7.119329303260822
GADES-GPU-dense	100	1000	100000	11382.3	4.056230027997624
amap	100	1000	100000	1147639.54	6.059805502721394
factoextra	100	1000	100000	105564423.72	8.02351758121579
pandas	100	1000	100000	4811953.186988831	6.682321393661297
pythonic	100	1000	100000	313599.5388031006	5.496375415315284
GADES-CPU-dense	1000	100	100000	13193718.26	7.120367205874967
GADES-GPU-dense	1000	100	100000	128568.35	5.109134070360473
amap	1000	100	100000	1390917.93	6.143301505549459
factoextra	1000	100	100000	104122127.9	8.017543035020994
pandas	1000	100	100000	280013845.3245163	8.447179505625718
pythonic	1000	100	100000	18998392.08126068	7.278716846227273
GADES-CPU-dense	10000	10	100000	33248760.88	7.521775464586309
GADES-GPU-dense	10000	10	100000	8921999.775	6.950462207953316
amap	10000	10	100000	10625947.64	7.026367671566651
factoextra	10000	10	100000	116798017.15	8.06743546993286
pandas	10000	10	100000	31340451537.53757	10.496105249275146
pythonic	10000	10	100000	1459172681.5223694	9.164106690244791
GADES-CPU-dense	10	100000	1000000	1482682926.35	9.171048286524323
GADES-GPU-dense	10	100000	1000000	3726815.575	6.571337901811641
amap	10	100000	1000000	287008169.625	8.457894259007718
factoextra	10	100000	1000000	10611946623.9	10.025795056896044
pandas	10	100000	1000000	801967.9069519043	5.90415698909143
pythonic	10	100000	1000000	191370.01037597656	5.281873880417331
GADES-CPU-dense	100	10000	1000000	1281668032.625	9.107775552274369

Supplementary Table 5. Adjusted R^2 values for linear models of logarithmic memory usage to log $|W|$ and logarithmic non-zero elements respectively.

Method	Counts	Non-zero elements
GADES-GPU-sparse	0.484	0.85
amap	0.998	-0.1
pandas	0.983	0.732
scipy	0.895	0.722
