# Supplementary material for: GPU-accelerated Kendall distance computation for large or sparse data
Source: Gigascience. 2024 Dec 9;13:giae088. doi: 10.1093/gigascience/giae088 (PMC11631066; doi:10.1093/gigascience/giae088)

GigaScience

GPU-accelerated Kendall distance computation for large or sparse data

--Manuscript Draft--

**Manuscript Number:**	GIGA-D-24-00103R3
**Full Title:**	GPU-accelerated Kendall distance computation for large or sparse data
**Article Type:**	Technical Note
**Funding Information:**	Russian Science Foundation (22-74-00023) — Mr Alexey Stupnikov
**Abstract:**	Background: Current experimental practices typically produce large multidimensional datasets. Distance Matrix calculation between elements e.g. samples for such data, although being often necessary in preprocessing for statistical inference or visualization, can be computationally demanding. Data sparsity (i.e. large number of zero elements), which is often observed in various experimental data modalities, such as single cell sequencing in bioinformatics or collaborative filtering in recommendation systems, may pose additional algorithmic challenges.<br>Results: We present GPU-Assisted Distance Estimation Software (GADES), a GPU-enhanced package that provides means for massively paralleled Kendall-τ distance matrices computation. Package's architecture incorporates specific memory management, which lifts the limits for the data size imposed by GPU memory capacity. Additional algorithmic solutions provide means to address data sparsity problem and reinforces the acceleration effect for large sparse datasets. Benchmarking against available CPU-based packages on simulated and on real experimental scRNA-seq or scATAC-seq datasets demonstrated significant speed boost for GADES for both sparse and dense data processing, with additional performance boost for the sparse data.<br>Conclusions: This work significantly contributes to the development of computational strategies for high-performance Kendall distance matrices computation and allows for the efficient processing of Big Data with the power of GPU. GADES is freely available at https://github.com/lab-medvedeva/GADES-main.
**Corresponding Author:**	Alexey Stupnikov, Ph.D.<br>Moscow Institute of Physics and Technology: Moskovskij fiziko-tehniceskij institut nacional'nyj issledovatel'skij universitet<br>Dolgoprudnyy, Moscow Region RUSSIAN FEDERATION
**Corresponding Author Secondary Information:**	
**Corresponding Author's Institution:**	Moscow Institute of Physics and Technology: Moskovskij fiziko-tehniceskij institut nacional'nyj issledovatel'skij universitet
**Corresponding Author's Secondary Institution:**	
**First Author:**	Pavel Akhtyamov
**First Author Secondary Information:**	
**Order of Authors:**	Pavel Akhtyamov
	Ausaaf Nabi
	Vladislav Gafurov
	Alexey Sizykh
	Alexander Favorov, PhD
	Yulia Medvedeva, PhD
	Alexey Stupnikov, PhD
**Order of Authors Secondary Information:**	
**Response to Reviewers:**	Following the Editorial Team's request, we have altered the style of code and data

Powered by Editorial Manager® and ProduXion Manager® from Aries Systems Corporation

	references in the 'Code availability' and 'Data availability' sections
**Additional Information:**	
**Question**	**Response**
Are you submitting this manuscript to a special series or article collection?	No
**Experimental design and statistics**<br><br>Full details of the experimental design and statistical methods used should be given in the Methods section, as detailed in our Minimum Standards Reporting Checklist. Information essential to interpreting the data presented should be made available in the figure legends.<br><br>Have you included all the information requested in your manuscript?	Yes
**Resources**<br><br>A description of all resources used, including antibodies, cell lines, animals and software tools, with enough information to allow them to be uniquely identified, should be included in the Methods section. Authors are strongly encouraged to cite Research Resource Identifiers (RRIDs) for antibodies, model organisms and tools, where possible.<br><br>Have you included the information requested as detailed in our Minimum Standards Reporting Checklist?	Yes
**Availability of data and materials**<br><br>All datasets and code on which the conclusions of the paper rely must be either included in your submission or deposited in publicly available repositories (where available and ethically appropriate), referencing such data using a unique identifier in the references and in the “Availability of Data and Materials” section of your manuscript.	Yes

Powered by Editorial Manager® and ProduXion Manager® from Aries Systems Corporation

Have you have met the above requirement as detailed in our Minimum Standards Reporting Checklist?	

Powered by Editorial Manager® and ProduXion Manager® from Aries Systems Corporation

LaTeX - Main Document (TeX file)
Click here to access/download;LaTeX - Main Document (TeX file);main.tex

```
This is pdfTeX, Version 3.141592653-2.6-1.40.26 (TeX Live 2024)
(preloaded format=pdflatex 2024.8.2)  15 OCT 2024 09:27
entering extended mode
 restricted \write18 enabled.
 %&-line parsing enabled.
**main.tex
(./main.tex
LaTeX2e <2024-06-01> patch level 2
L3 programming layer <2024-05-27>

! LaTeX Error: File `oup-contemporary.cls' not found.

Type X to quit or <RETURN> to proceed,
or enter new name. (Default extension: cls)

Enter file name:
! Emergency stop.
<read *>

l.11 ^^M

*** (cannot \read from terminal in nonstop modes)


Here is how much of TeX's memory you used:
 19 strings out of 473583
 444 string characters out of 5732343
 1925908 words of memory out of 5000000
 23012 multiletter control sequences out of 15000+600000
 558069 words of font info for 36 fonts, out of 8000000 for 9000
 1141 hyphenation exceptions out of 8191
 19i,0n,29p,95b,17s stack positions out of
10000i,1000n,20000p,200000b,200000s
!  ==> Fatal error occurred, no output PDF file produced!
```

Manuscript

Click here to access/download;Manuscript;GADES_v3.pdf

OXFORD

(GIGA)^n SCIENCE

GigaScience, 2017, 1–10

doi: xx.xxxx/xxxx
Manuscript in Preparation
Paper

PAPER

GPU-accelerated Kendall distance computation for large or sparse data

Pavel Akhtyamov[1,2,†], Ausaaf Nabi[1,2], Vladislav Gafurov[1,2], Alexey Sizykh[1,2,3], Alexander Favorov[4,5], Yulia Medvedeva[1,2,6] and Alexey Stupnikov[1,2,*]

[1]Department of Biomedical Physics, Moscow Institute of Physics and Technology, 141701, Dolgoprudny, Russia and [2]Moscow Center for Advanced Studies, 20, Kulakova Str., Moscow, Russia. and [3]Department of Biochemistry and Medical Genetics, University of Manitoba, Winnipeg, MB R3E 3P5, Canada, and [4]Johns Hopkins University School of Medicine, Baltimore, MD 21205, USA, and [5]Vavilov Institute of General Genetics, Moscow, 119333, Russia, and [6]Research Center of Biotechnology,117312, Moscow, Russia

[†]akhtyamovpavel@gmail.com
[*]aleksej.stupnikov@phystech.edu

Abstract

Background: Current experimental practices typically produce large multidimensional datasets. Distance Matrix calculation between elements or samples (*e.g.* samples) for such data, although being an often used type of necessary in preprocessing or for statistical inference or visualization, can be computationally demanding. Data sparsity, which is often observed in various experimental data modalities, such as single cell sequencing in bioinformatics or collaborative filtering in recommendation systems, may pose additional algorithmic challenges.
Results: We present GPU-Assisted Distance Estimation Software (GADES), a GPU-enhanced package that allows for massively paralleled Kendall-τ distance matrices computation. Package's architecture involves specific memory management, which lifts the limits for the data size imposed by GPU memory capacity. Additional algorithmic solutions provide means to address data sparsity problem and to reinforce the acceleration effect for sparse datasets. Benchmarking against available CPU-based packages on simulated and on real experimental scRNA-seq or scATAC-seq datasets demonstrated significantly higher speed for GADES compared to other methods for both sparse and dense data processing, with additional performance boost for the sparse data.
Conclusions: This work significantly contributes to the development of computational strategies for high-performance Kendall distance matrices computation and allows for the efficient processing of Big Data with the power of GPU. GADES is freely available at https://github.com/lab-medvedeva/GADES-main

Key words: Kendall correlation; distance matrix; GPU; parallel computation; high dimension; scRNAseq; scATACseq.

Introduction

Experimental data often consist of numerous multidimensional objects. It is hard for a single data point to carry sufficient information for understanding the nature of the underlying process (AKA the physical sense). Instead, the massive set of input points provides meaningful information after being classified, or assigned to clusters, or joined into trajectories.

Current experimental practices typically produce large arrays of multidimensional points. Extensive set of these input elements can provide meaningful information after the elements have been assigned to clusters, or joined into trajectories [1, 2]. All these procedures require pairwise distances between the data points to find neighbouring elements with similar properties. Such procedures require computation of pairwise distances between elements. Among metrics routinely used for this task is Kendall-τ rank-based correlation coefficient [3], which is intensively applied in various fields, such as recommender systems [4, 5], bioinformatics [6, 7], graph

Compiled on: October 15, 2024.
Draft manuscript prepared by the author.

Key Points

- We present GADES, the first package for GPU-accelerated massively paralleled computation of Kendall distance matrices
- Special memory manipulation approach allows to bypass data size limits imposed by GPU memory capacity, and allows one to process large datasets with GPU acceleration
- Specific algorithmic implementation gives an option to process sparse matrices (e.g. scRNA-seq or scATAC-seq data) with additional acceleration
- GADES outperforms available packages regardless of data size, when benchmarked (both in sparse mode or dense mode) on simulated and real experimental scRNA-seq or scATAC-seq datasets

and network analysis [8, 9] due to the metric's high reliability and robustness [10, 11, 12].

~~Since the data points are numerous and they exist in a highly multidimensional space, the distance matrix calculation poses a large set of simple computational tasks.~~ Large number and high dimensionality of the data points turn the calculation of the distance matrix into a massive set of simple computational tasks. The pairwise distance computation, thus, ~~appears to be of~~ has high computational cost, and ~~it~~ may be the bottleneck of ~~all~~ the data analysis [13]. ~~Nevertheless~~At the same time, some Kendall-τ distance calculation algorithms are suitable for parallelisation~~, and even if a particular pairwise calculation is hopelessly sequential, it is natural to run the procedure in parallel for different point pairs. Particularly, the parallelisation can be implemented by recruiting GPU instead of CPU.~~, particularly, the parallelisation can be implemented by recruiting GPU instead of CPU. ~~Additional~~Another important property experimental datasets often possess is high-degree sparsity (i.e. large number of zero elements in experimental data). This quality is notably common in regards to the single cell data modality in bioinformatics [14]. Albeit often considered a problem [15, 16, 17], this data characteristic also may grant additional benefits for some analysis types [18], potentially including the Kendall-τ distance matrix generation. Although GPU-accelerated distance metrics computation for other metrics was recently introduced [19], no such option has been available for Kendall-τ distances yet.

In this manuscript, we present GADES (GPU-Assisted Distance Estimation Software) that allows for the GPU-accelerated pairwise Kendall-τ distance calculation for large datasets of highly multidimensional points. GADES can be run in a parallel paradigm using the existing CUDA framework for GPU calculations, with a supplementary option to algorithmically take advantage of data high sparsity. We further demonstrate its application to a set of simulated and real experimental scRNA-seq and scATAC-seq datasets.

Motivation

Kendall distance between two distribution vectors is calculated as follows. Given vectors $x = (x_i)_{i=1}^{F}$ and $y = (y_i)_{i=1}^{F}$, the number of discordant pairs D is calculated. The pair $(i, j), 1 < i < j \leq F$ is called discordant, whether $(x_i - x_j) \cdot (y_i - y_j) < 0$. Therefore, the distance formula is $\tau(x, y) = 2 \cdot \frac{D}{F \cdot (F-1)}$. In other words, Kendall distance shows the normalized number of pairs, which order in x and y differs.

Several CPU-based packages for calculating Kendall distances between vectors were previously introduced. They can be divided into the two groups: the packages of the first group compute pairwise distances of the input matrices columns and includes packages in R, such as amap [20] and factoextra [21]. The second group of packages is implemented in Python (scipy [22] and pandas[23]), and only supports vector-by-vector distances calculation; input matrices, therefore, need to be split by columns to produce distance matrices (Table 1).

The amap package was initially designed for the principal component analysis and clustering inference. Its main application is the parallel computation of distance matrices. The package factoextra provides means for the most complex set of tools for the component analysis and results visualization, however, it does not support parallel computations. The scipy package allows for the basic vector and tensor manipulations with the backend enhanced with C libraries. It calculates the Kendall distance employing Fenwick tree[24, 25],which is less computationally demanding than the pairwise inversion calculation. Pandas package allows for various statistical manipulations, including Kendall distance calculation between pairs of vectors. However, the package does not support parallel computations.

Implementing GPU-delivered massive parallelization appears to be a natural solution to accelerate Kendall distance matrix computation. In endeavor to approach this challenge, two aspects need to be taken into consideration. First, GPU hardware is known to have limited RAM capacity, that reduces amount of data can be handled in one run, which implies severe restraint on the input matrices size. To bypass this restriction and to allow for large matrices to be processed, a specific memory manipulation procedure needs to be introduced to split the input data into portions (that we refer to as *batches*) that fit properly into GPU RAM. Second, GPU initialization overhead increases time of computation process, which can have a significant affect on the small datasets processing efficiency. Therefore, an option of employing CPU instead of GPU for small matrices is required.

Taking into account high degree of the input matrices sparsity may reduce the required number of algorithm's operations by omitting certain procedures with zero elements and, consequently, reinforce the boost in computational speed. Therefore, for the sparse input data an additional processing option can be implemented (that we refer to as *sparse* mode, in opposition to *dense* mode, that makes no account for data sparsity), allowing to take advantage of this data type specific structure.

Finally, for many commonly employed metrics the computation procedure requires multiple matrices' multiplication procedures before an aggregation step (e.g. taking square root of the sum of three matrices multiplications for the Euclidean metric), which fits naturally in the most GPU-accelerated libraries standard methods. Kendall distance computation, however, needs the detection of pair being a discordant step followed by computationally demanding aggregation step (calculating a sum of the discordant pairs detected by each pair of the features) at an earlier stage, and, therefore, cannot be transduced to the matrix multiplication without a significant loss in algorithm's efficiency. Therefore, the proposed approach, when implemented in GPU parallelization paradigm with no matrices' multiplication, will require additional algorithmic solutions for both sparse and dense modes.

Guided by these considerations, we sought to design GADES package, that provides calculation of Kendall-τ distance with the following features:

- an option to choose between GPU and CPU hardware for acceleration of the distances computation,

- processing input data in batches of particular size to fit into the RAM of a given GPU setup,
- an option to select sparse mode or dense mode for the distance matrix calculation.

Methods

Algorithm Description

Given matrix M of size $C \times F$ (to distinguish between the two dimensions of M we refer to them as *Features* and *Cells*) we calculate Distance Matrix DM of the size $C \times C$ (thus, computing distances between *cells*). Element $D_{i,j}$ is calculated using elements $DM_{i,j} = \tau(M_{i*}, M_{j*})$, where *tau* is the Kendall correlation distance, M_{i*} denotes the corresponding row of the matrix (Figure 1A). Since the elements are calculated independently, their calculation can be parallelized.

As Distance Matrix calculation requires additional memory for storing intermediate calculations, DM needs to be calculated separately using batches as follows. We split matrix M into N matrices $\{M_t\}_{t=1}^{N}$ of the size $B \times F$ where B could be selected by the user. For each pair batches (m, n) we select rows (k, p) respectively and store the values in the intermediate metric tensor structure $TM^S_{mB+k,nB+p}$ where $S \in [0; F-1] \times [0; F-1]$ for Kendall correlation. The element of distance matrix $DM_{mB+k,nB+p}$ is calculated in parallel by k and p using the stored $TM^S_{mB+k,nB+p}$ values (Figure 2A). Since we use the `atomicAdd` operation, which works with the speed of L2 cache, to aggregate values of TM, we do not use any of shared memory or alternative caching techniques. It is quite a common case that the input data matrix is sparse. These sparse input matrices challenges the computation of the DM to moderate the memory usage by omitting calculation for the missing elements. In addition, distance calculation should treat missing values as zeros, therefore, each element should be accounted whether at least one of $M_{k,S}$ or $M_{p,S}$ is presented in the Sparse Matrix). We use the sweeping line method: for every pair of *non-zero* elements $M_{k,S}$, $M_{p,S}$, where $S = (u, d)$, $u \leq d$, let us denote $M_{l,u}$ the first non-zero element before $M_{k,u}$ and $M_{r,u}$ the first non-zero element after the $M_{p,u}$. During calculation of $TM^S_{k,p}$ the method also calculates $TM^S_{i,p}$ for i in range (l, k) and $TM^S_{k,j}$ for j in range (p, r) (Figure 1C, Figure 2B). Thus, GADES precisely identifies all quadruples of non-zero elements $M_{i,u}$, $M_{i,d}$, $i \in (l, k)$ and $M_{j,u}$, $M_{j,d}$, $j \in (p, r)$ calculating indices of row elements which belongs to corresponding intervals (Figure 2B).

In other words, the calculation of all one-zero elements happens at the same time with their common neighbouring non-zero pair, thus, reducing computational time as the complexity reduces from the number of all pairs to the number of non-zero pairs. This allows GADES to increase the speed quadratically to the density of the input count matrix. Sparse matrices are loaded as COO (Coordinate list) format and converted into the CSR (Compressed Sparse Row) format [26] to achieve sequential scan over the indices in the sweeping line method.

Data Description

Datasets of three types were recruited to evaluate packages' performance. First, sets of simulated dense matrices of various size were created to explore the effects of data dimensionality. Second, simulated sets of matrices with fixed sparsity were generated to test methods on sparse data. Third, real experimental single-cell RNA-seq and ATAC-seq datasets were employed to observe methods' efficiency on real experimental data.

Simulated dense data

To explore packages' performance we generated 9 sets of simulated integer matrices of different sizes with fixed random seed for experiments reproducibility. The dimensions varied from 10^2 to 10^5 (Supplementary table 1). Small matrices were employed to estimate the overhead for GPU usage whereas large matrices were used to explore the limits where CPU-based approaches could not cope with the computational task. The sparsity of data was not taken into account for these datasets, to which we refer to as *dense*.

Simulated sparse data

To evaluate packages' performance on sparse data, we simulated sparse datasets by inserting a number of zero elements into previously generated dense matrices. This resulted in a set of matrices of the same size as the dense matrices, and sparsity degree varying from 0.5 to 0.99. (Supplementary table 2).

Experimental single cell data

Fourteen experimental single cell datasets were recruited to evaluate packages' performance for real data. To take into account the dimensionality effect we employed scRNA-seq ($N_{features} \sim 10^4$) and scATAC-seq ($N_{features} \sim 10^5$) datasets.

PBMC3K dataset [27] comprises of scRNA-seq data of 2700 Peripheral Blood Mononuclear Cells. We have extracted 3 subsets of this dataset: complete matrix (2700 cells), B and T cells (1806 cells), B and CD8T Cells (623 cells). Human Lung Cell Atlas [28] contains UMI-based data of mixed blood and lung cells. We extracted three tissue-wise subsets: bone marrow (5037 cells), aorta (408 cells), lung (1716 cells). Fibroblasts and Cardiomyocytes Dataset [29] comprises of scRNA-seq and scATAC-seq data for reprogramming cells from fibroblasts to cardiomyocytes. scRNA-seq dataset consists of 27999 cells and 26124 genes; scATAC-seq count matrix contains 79514 cells and 287000 peaks. HumanCortex dataset [30] contains gene expression programs of fetal neocortex development with 734 cells and 18927 genes. MouseHypothalamus dataset [31] consists of Drop-seq based scRNA-seq of the mouse hypothalamus cell development with 14437 cells and 23284 features. HSC dataset [32] contains scATAC-seq data of hematopoiesis development for 2034 cells and 234000 peaks. TCells dataset [33] consists the scATAC-seq data of T Cells proliferation with 765 cells and 49345 peaks. CellLines dataset [34] contains the scATAC-seq data used to detect the trans-factors of cell-to-cell interactions with 1224 cells and 125648 peaks. The summary for the datasets is presented at Supplementary table 3.

Evaluation of performance

To evaluate GADES' performance, ~~it was tested~~ we tested it against available non-GPU packages for Kendall distance computation: factoextra [21], amap [20], scipy 1.10.1 [22], pandas 1.5.3 [23]. Package amap was run with CPU acceleration, packages factoextra, pandas and scipy were run without hardware acceleration.

On the dense simulated datasets GADES was run in two setups: one - with GPU acceleration in dense mode, second - with CPU acceleration in dense mode (that we refer to as *GADES-GPU-dense* and *GADES-CPU-dense* respectively). On the sparse simulated and real experimental datasets both the hardware acceleration type (GPU/CPU modes), and the accounting for data sparsity (sparse/dense modes) were varied, thus, resulting in four GADES setups (*GADES-GPU-dense*, *GADES-CPU-dense*, *GADES-GPU-sparse* and *GADES-CPU-sparse*).

All the packages were deployed at the HPC cluster with NVIDIA RTX 3090 with 24GB VRAM and 24 CPUs available. We used cores without Hyperthreading or Virtualization mechanisms enabled. All the experiments were run with same setup: 1 GPU for GADES and 24 CPUs for multiprocessing packages (amap); if the package did not support multi-threading, we used 1 CPU (Table 1). To ensure

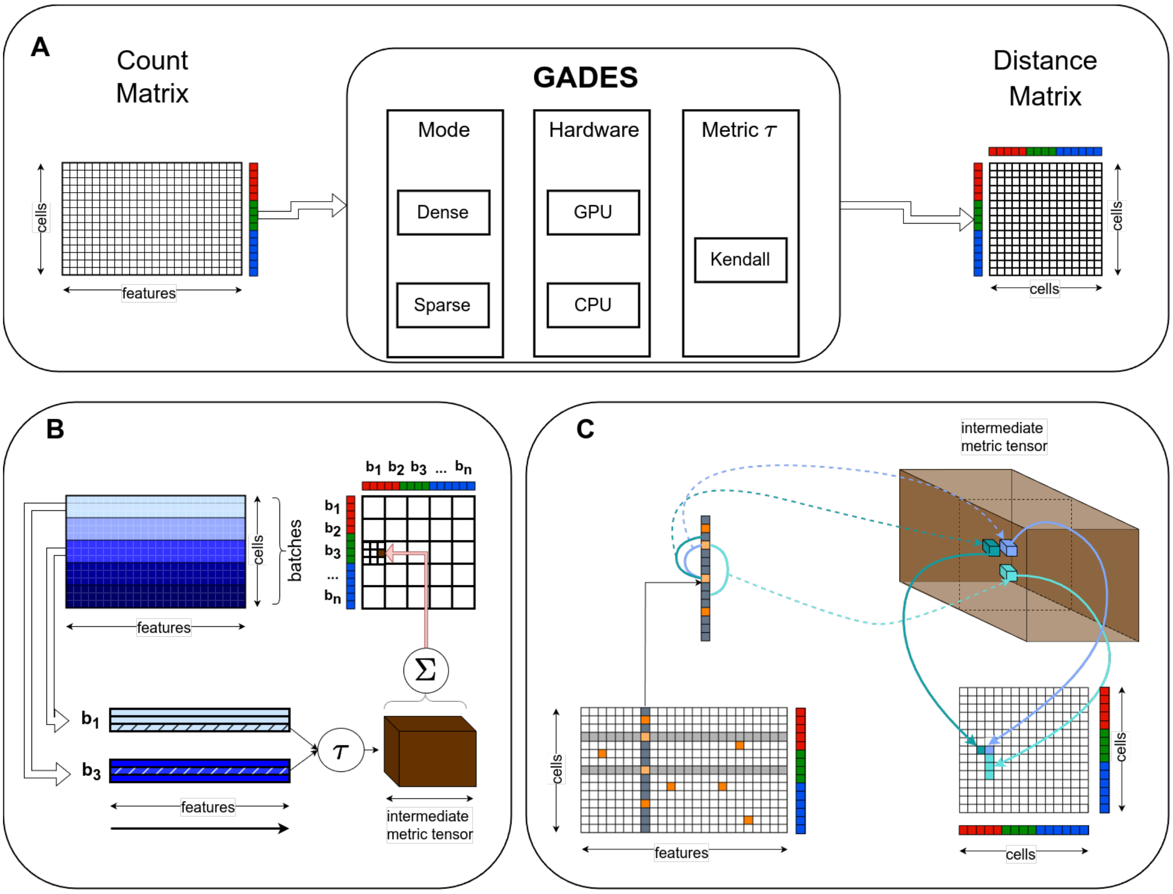

Figure 1. GADES pipeline overview a) GADES general framework. Given Count Matrix with C cells and F features, GADES calculates Kendall distance matrix using Dense or Sparse mode on GPU or CPU hardware. b) GADES batch-wise Count Matrix processing. Count Matrix $C \times F$ is splitted into batches of size $B \times F$ each. Distance matrices between 2 batches are calculated in parallel for the features. The results are accumulated using `atomicAdd` CUDA operation. Then, block matrices of size $B \times B$ that correspond to each batch are concatenated into the final distance matrix. c) GADES distance matrix computation with sparse layout for specific row. One pass of distance calculation combines calculation of pairs of non-zero elements and sliding of one-zero range pairs.

Table 1. Overview of packages applicable for Kendall distance matrices computation and their setup in benchmarking

Package	Language	Kendall	CPU mode	CPU Multicore	GPU mode	Benchmarking setup
amap	R	τ_a	Yes	Yes	No	24 CPU threads
pandas	Python	τ_a	Yes	No	No	1 CPU thread
scipy	Python	τ_a	Yes	No	No	1 CPU thread
factoextra	R	τ_b	Yes	No	No	1 CPU thread
GADES	R/CUDA	τ_a	Yes	No	Yes	10496 CUDA cores in GPU mode<br>1 CPU thread in CPU mode

amap method employed physically all the allocated cores (24, see above) rather than approximately 1/2 that would be expected for a virtual parallelization setup, we measured the average CPU usage for the 24 threads and observed the CPU core usage being at the level of 2200 % of a single CPU performance. Since Kendall distance computation, differing from other metrics, cannot not be reduced to matrix multiplication, implicit built-in parallelization in some libraries was not set and the number of processes for the packages was specified explicitly.

We applied all the packages to a number of simulated and real single-cell datasets (See Data Description for details). Each matrix was processed with each method 25 times to take processor throttling into account; every real dataset was processed with every method 10 times. Time limit for every run of a method was set up at 1 day (86 400 seconds). For all the experiments computational time $T_{package}$ and relative *acceleration* as a scaling factor $A = \frac{T_{package}}{T_{GADES-GPU-dense}}$ of a package compared to GADES-GPU-dense running time. The results visualization was plotted with seaborn package [35].

Evaluation of optimal batch size

To assess the effect of the batch size on the performance of GADES-GPU method, we applied the following procedure. All the analyzed datasets were additionally processed with GADES-GPU-dense method for generated dense datasets and with GADES-GPU-sparse method for generated sparse and experimental single cell datasets. For generated datasets we selected batch sizes being divisors of the cells number, namely, 100, 500, 1000, 5000 and 10000 cells per batch. For the experimental single cells we selected batch sizes of 250, 500, 1000, 2000 and 4000 cells.

As the optimal performance was achieved at batch size 500 cells (see Results), we represented the performance for each analyzed batch size B as the performance factor $A_B = \frac{T_{500}}{T_B}$, where T_{500} is the computational time for a batch size of 500 cells.

Evaluation of the memory usage

To explore the RAM usage of all the tested packages, including

A

```
Algorithm 1: GADES algorithm for dense matrices
Data: Dense matrix M of size C × F;
Disc - discordant function for Kendall correlation
Result: DM of size C × C
parallel for S = (u, d); 0 ≤ u ≤ d ≤ F − 1 do
    for 0 ≤ k < p < C do
        atomicAdd(DM_{k,p}, Disc(M_{k,S}, M_{p,S}));
    end
end
```

B

```
Algorithm 2: GADES algorithm for sparse matrices
with Kendall distance
Data: Sparse matrix M of size C × F: contains of
      indices, values - lists of F elements;
Disc - discordant function for Kendall correlation
Result: DM of size C × C
parallel for S = (u, d); 0 ≤ u ≤ d ≤ F − 1 do
    col_count = # existing elements in M_{*,u};
    for 0 ≤ col1_index ≤ col2_index < col_count do
        l = indices[u][col1_index - 1];
        k = indices[u][col1_index];
        p = indices[u][col2_index];
        r = indices[u][col2_index + 1];
        M_{k,u} = values[u][col1_index];
        M_{p,u} = values[u][col2_index];
        move indices to range M_{a,d} for a ∈ (l, k) ∪ (p, r)
         with sweeping line;

        detect values of M_{a,S} for a ∈ (l, k) ∪ (p, r) with
         discovered indices;

        atomicAdd(DM_{k,p}, Disc(M_{k,S}, M_{p,S}));
        for l < a < k do
            atomicAdd(DM_{a,p}, Disc(M_{a,S}, M_{p,S}));
        end
        for p < a < r do
            atomicAdd(DM_{k,a}, Disc(M_{k,S}, M_{a,S}));
        end
    end
end
```

Figure 2. GADES algorithms pseudocode A) for the dense mode B) for the sparse mode.

GADES, we employed memory profiling tools: profmem package [36] for the R language and with psutil package [37] for the Python language. We measured the peak memory usage by disabling the implicit calls of the garbage collector. To avoid extra calculations, we subtracted the allocated memory size, which is required for storing the obtained distance matrix, from the result. For the GADES method, we tested all the modes with the batch size 500 that was shown to be optimal batch size for the computational time.

Results

To evaluate the performance of the packages we computed their relative acceleration compared to GADES-GPU-dense running time $A = \frac{T_{package}}{T_{GADES}}$, thus, using GADES-GPU-dense as baseline (see Methods: Evaluation of performance). $\log A < 0$ implies the package runs more slowly than GADES-GPU-dense on the same data, $\log A > 0$ means package demonstrates higher efficiency compared to GADES-GPU-dense. For cases when the time limit was reached and the computation failed, the results computation time was set to the value of time limit of 86400s.

Case study: simulated dense data

We explored the patterns of acceleration for the three sets of dense matrices depending on their size $|W| = N_{features} \times N_{cells}$ ($|W| \in \{10^5, 10^6, 10^7\}$) (see Methods: Data description).

GADES-GPU-dense demonstrated significantly higher data processing speed than the rest of the packages and all $|W|$ values (Figure 3A, Supplementary Figure 1). The worst performance was shown by factoextra and GADES-CPU-dense, which run 2800 and 340 times more slowly, than GADES-GPU-dense. The efficiency of scipy and pandas was higher, and their speed is 120 and 75 times lower, than the speed of GADES-GPU-dense. These packages' performance also moderately improves with the increase of $|W|$, due to the details of their asymptotic complexity [23, 22].

Some boxplots displayed significant level of variance across processing time for matrices with the same $|W|$, delivered by different combinations of dimensions N_{cells} and $N_{features}$, which may imply an unequal impact of dimensions (N_{cells} and $N_{features}$) increase on the running speed. To examine the magnitude of this effect we analyzed separately the running time observed for each (N_{cells}, $N_{features}$) pair in the processed datasets (Figure 3B). For GADES-GPU-dense, scipy and pandas packages increase in N_{cells} affects the running time more intensely, then in $N_{features}$. Differently, for amap package, the impact of $N_{features}$ increment is stronger than the one of N_{cells}. For GADES-CPU-dense and factoextra, the influence of N_{cells} and $N_{features}$ augmentation proved to be comparable.

Case study: simulated sparse data

For the simulated datasets of varied sparsity degree (see Methods: simulated sparse data for details), we have observed no effect of sparsity on the performance of existing CPU-based packages, as well as on GADES-CPU-dense and GADES-GPU-dense (Figure 4, Supplementary Figure 2). GADES-CPU-sparse and GADES-GPU-sparse, in turn, demonstrated significant raise in running speed with the increase of input data sparsity. The magnitude of the effect augments with the size of the input data $|W|$ increment. Thus, GADES-CPU-sparse runs 6.8 times more quickly than the GADES-CPU-dense baseline for $|W| = 10^5$, 14 times more quickly for $|W| = 10^6$, and 16 times more quickly for $|W| = 10^7$. For the degree of sparsity 0.99, GADES-CPU-sparse performance becomes comparable to the one of GPU-dense, as GADES-CPU-sparse runs 520 times more quickly than GADES-CPU-dense for $|W| = 10^6$, and 850 times more quickly for $|W| = 10^7$.

Case study: experimental single cell datasets

Run on real single cell datasets, packages demonstrated high failure rate (i.e. number of datasets failed to be processed within the timelimit) (Figure 5A). Four datasets (PBMC5K, MouseHypothalamus, Fibrocard-RNA, Fibrocard-ATAC) out of 14 could not be processed within the time limit by any of the packages. The only exception is GADES-GPU-sparse, that managed to process two of them (MouseHypothalamus, Fibrocard-RNA) and had the lowest failure rate across all the packages. The two datasets that all the packages failed to process were excluded from the further analysis.

For the remaining properly-processed datasets GADES-GPU-sparse proved to be the most efficient approach (Figure 5B). Its running speed was 7 times higher than GADES-GPU-dense baseline. The pandas package showed reasonable performance compa-

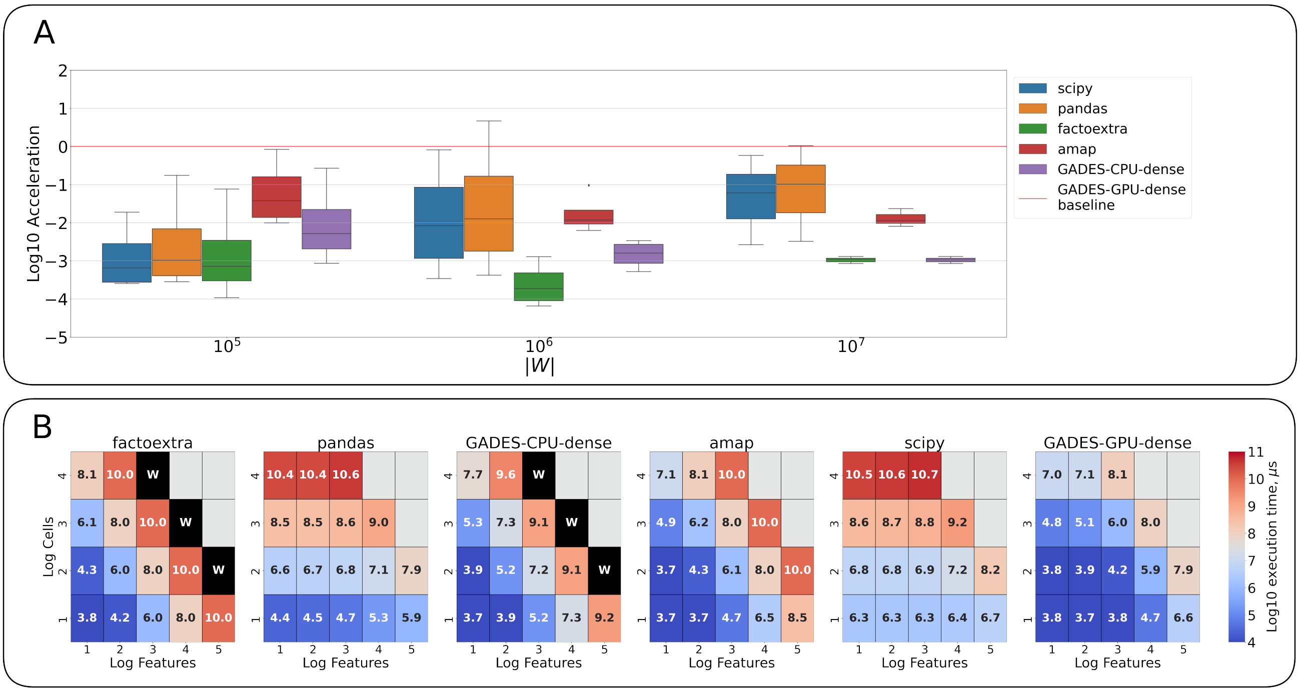

Figure 3. Benchmarking results for dense generated datasets and all the benchmarked packages. A) Log-scaled packages acceleration for different input matrix size |W| in comparison with GADES-GPU-dense processing time as the baseline. B) Heatmaps of the Log-scaled mean computation time for every set of dimensions (cells and features); results for matrices failed to process in 24 hours walltime marked W.

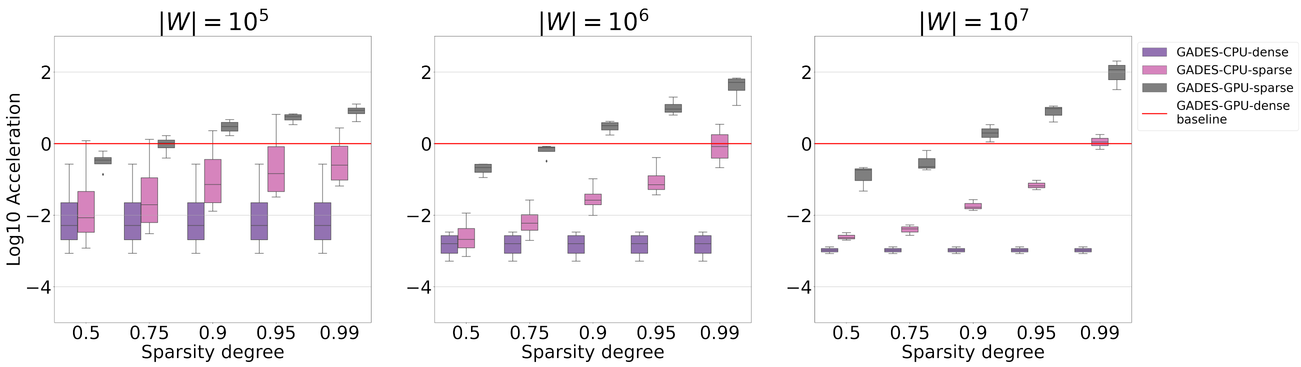

Figure 4. Benchmarking results for sparse generated datasets and all GADES modes: the impact of the data sparsity degree on Log-scaled acceleration in comparison with GADES-GPU-dense processing time as the baseline.

rable to the baseline (although 12 % lower), yet run 7.8 times more slowly than GADES-GPU-sparse. All other packages demonstrated lower speed than baseline (from 21 times more slowly for factoextra package, to 2.7 times decrease in speed for scipy), Thus, GADES-GPU-sparse displayed the best efficiency both in terms of number of processed (i.e. not failed due to the time limit) datasets, and the speed of their processing.

Computational cost Study: GPU efficacy dependence on the batch size

For the real and generated datasets, package GADES-GPU demonstrates the same pattern of the efficiency dependence on the batch size (Figure 6). For the generated sparse and real datasets the optimal batch size is typically 500 cells. For the real datasets the GPU efficacy performance factor (see Methods) for the batch size of 250 can be more than 1 or less than 1, and it depends on the input matrix, whereas for the generated sparse dataset the lower batch size it is robustly more than 1. At the same time, for the generated sparse datasets the factor for the batch size of 1000 cells could be more or less than 1 (Figure 6A, Supplementary Figure 3). Therefore, the most stable results are observed for the batch size of 500, which is recommended for the default parameter for analysis, as it can deliver reasonable trade-off between cost of context switching for lower batch sizes and computationally challenging interactions while calculating distance matrix for the same batch.

Computational cost Study: Memory usage

In respect to the memory usage, we observed differing patterns for Python-based and R-based packages, as Kendall correlation for the scipy and pandas packages are calculated in-place. Among the R packages, GADES-GPU-dense package has the lowest memory usage (Figure 6B). Furthermore, an increase in the degree of sparsity for the generated sparse datasets was associated with redaction in memory usage for by GADES-GPU-sparse method, and the memory usage heavily depended on the non-zero elements count. To affirm the observation, we measured RAM usage of GADES-GPU-sparse for all real datasets using batch size of 500 elements. We fitted model $\log(Memory) \sim \log(NonZeroElements)$. Surprisingly, we observed the explained variability $R^2 = 0.85$, while for other benchmarked packages the values were lower (0.72, −0.1, 0.73 for

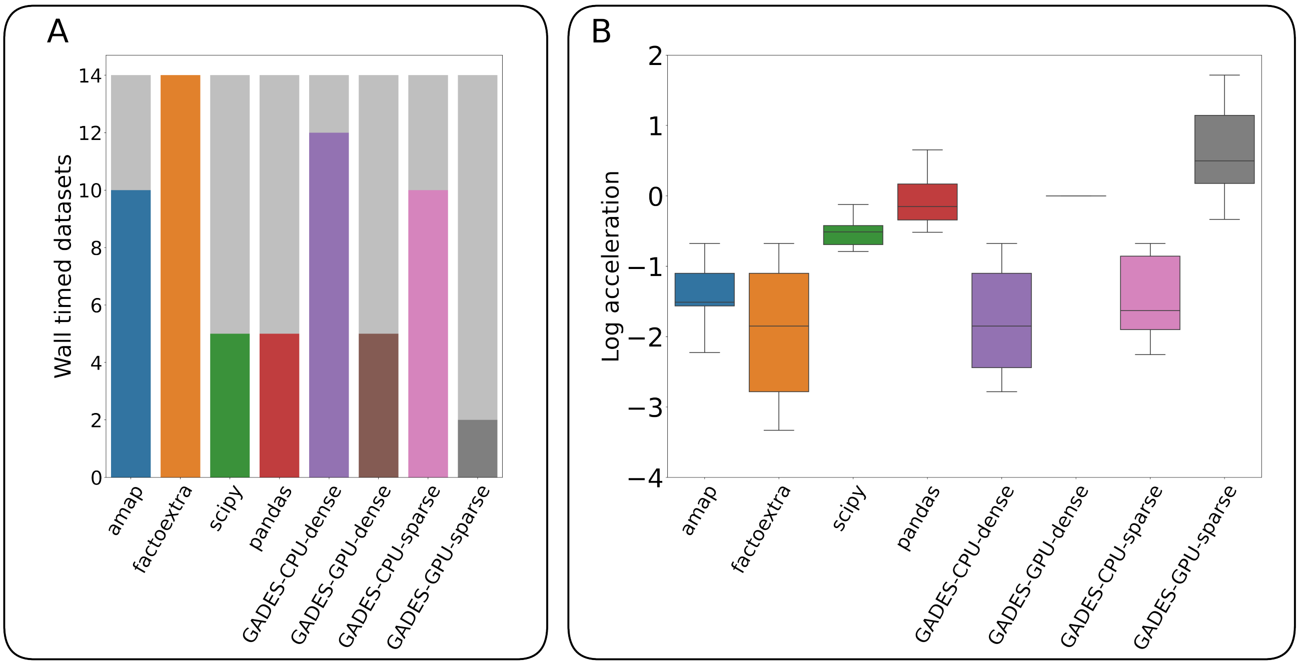

Figure 5. Benchmarking results for the real experimental datasets. A) Number of datasets failed to process within the running time limit we set for all the packages. B) Log-scaled acceleration for all the packages in comparison with GADES-GPU-dense processing time as the baseline

scipy, amap and pandas packages correspondingly) (Supplementary Figure 4, Supplementary Table 5). Thus, regarding the memory usage, the GADES-GPU package is comparable to CPU-based packages for the Kendall distance matrix calculation in dense mode and has a memory advantage in sparse mode.

Discussion

In this study, we have presented GADES, the first computational package to compute Kendall-τ distance matrices ~~with~~ using the advantage of the massively parallel computation delivered by the GPU acceleration. The implemented specific memory management lifts the limits for the data size imposed by GPU memory capacity, allowing processing datasets of any size. In addition, the algorithmic realization of the package's architecture allows to take advantage of the input data sparsity, providing additional boost to the computational performance. GADES can be run on either CPU or GPU hardware, that makes it flexible for various computational architecture setups and allows for optimal processing of datasets of different size.

For the simulated dense datasets we have observed GADES in GPU and dense mode to drastically outperform all other packages regardless of the datasets size and running 75 times more quickly than pandas, its nearest rival. For the simulated datasets with high degree of sparsity (more than 75%-90% zeroes in dataset) GADES in GPU and sparse-aware mode outperforms other packages acutely regardless of the datasets size, with a tendency to better performance for larger size of the input data or higher degree of sparsity. However, the effect observed for the real scATAC-seq and scRNA-seq datasets is less radical, although GADES with GPU acceleration in sparse-aware mode still performs computations significantly more quickly than the rest of the packages (7.8 times more quickly than the second best package). This displays the effect the structure of input data, including non-random distribution of zero elements in the input matrix, may have. In terms of RAM resources efficiency, Python-based packages showed better performance compared to R-based packages for dense data, which is not surprising since Python packages handle `float` arrays, whereas R packages work with `double` data type; additionally, Python-based packages perform operations in-place. GADES-dense mode proved to be comparable to other R packages in RAM resources efficiency, and showed significant boost in performance for sparse mode.

Kendall-τ correlation, being a robust non-parametric similarity measure, is widely employed for multidimensional data analysis in cases when the normality propositions are weakly based. However, its applicability is often limited, especially in Big Data domain, by the high computational costs and unreasonable running time. The GADES package, the first method with GPU acceleration and batched memory manipulation and algorithmically boosted for highly sparse data, lifts these constraints. This makes GADES a powerful tool giving means to broadly and efficiently apply the Kendall-τ correlation to large datasets in numerous engineering and scientific fields.

Current realization of GADES has several limitations. First, for GADES in GPU mode it is the necessity for data transfer into GPU RAM, that CPU-based packages naturally are free from. This may have a negative impact on the package's running time for the datasets of smaller size, however, it can smoothly be addressed by falling to CPU mode of GADES. Second, GADES-GPU may demand more RAM resources for data processing compared to CPU-based packages in some scenarios. In addition, current implementation of GADES lacks option for the parallelization in CPU mode, which for some benchmarking setups allowed other CPU-based packages to be more efficient than GADES-CPU.

One of the main directions for extending GADES is altering method's architecture to introduce CPU parallelization, that will improve the efficiency of low dimensionality data processing. Incorporating procedures to calculate additional distance types, such as Euclidean, Spearman or cosine distances, is one more avenue to improve the functionalities of the package. Another task to address is enhancing the versatility of GADES approach via transferring the designed computational core to alternative platforms, such as introducing Python version of GADES package.

The presented package, GADES, allows for more efficient large dimension or sparse data preprocessing including, but not limited to, the results of the single cell NGS experiments, e.g. scRNA-seq and scATAC-seq. The package provides means to improve efficiency, sensitivity, and running speed of the existing analysis practice in bioinformatics and other ~~fields~~ areas.

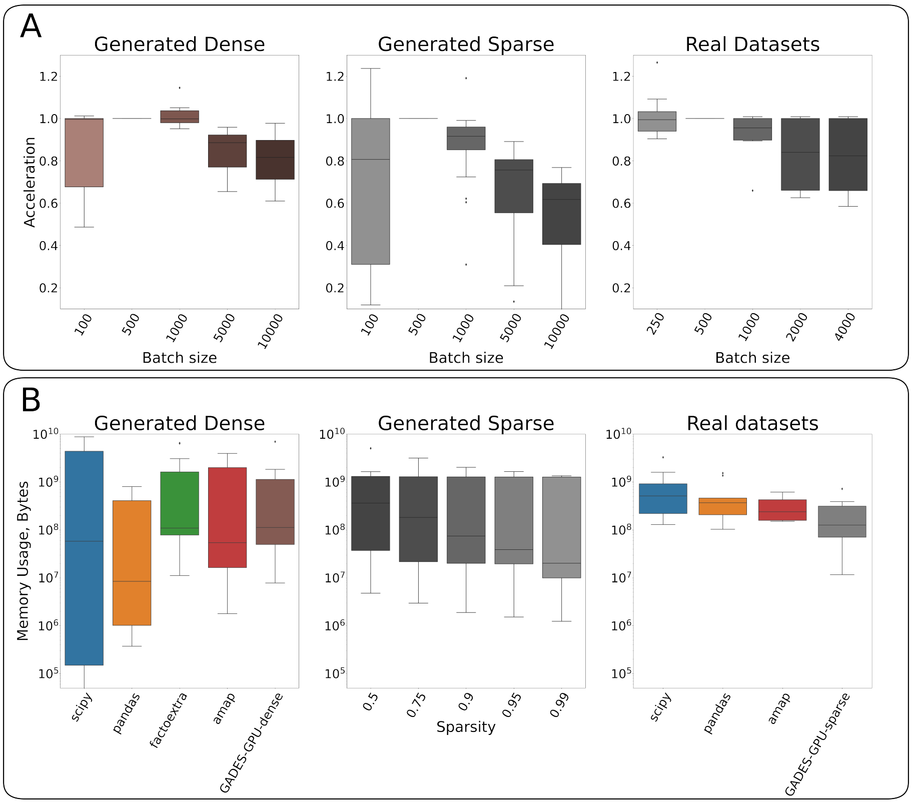

Figure 6. Memory usage and the effects of batch size. A) GADES performance (in dense mode for generated dense datasets, and in sparse mode for generated sparse and real datasets) for all the datasets and various batch sizes with baseline of the batch size of 500 elements. B) Memory usage of all the tested methods for the generated dense and real datasets, and GADES-sparse for the generated sparse datasets.

Availability of source code and requirements

GADES source code
Project name: GADES-main
License: GPL-3.0 license
Project home page: `https://github.com/lab-medvedeva/GADES-main`
PID: swh:1:snp:8550b8daa79a0ca2b0ce11dba2c6e9f880fb4009
Citation: Akhtyamov, P., Nabi,A., Gafurov,V., Sizykh,A., Favorov,A., Medvedeva,Y., Stupnikov,A. "GADES - GPU-Assisted Distance Estimation Software" [Computer software]. Software Heritage, `https://archive.softwareheritage.org/swh:1:snp:8550b8daa79a0ca2b0ce11dba2c6e9f880fb4009;origin=https://github.com/lab-medvedeva/GADES-main`
Operating system(s): Platform independent
Programming language: R 4.1+, CUDA 11+
Other requirements: see public environment file
RRID: SCR_02551

GADES reproducibility code
Project name: Article-GADES
License: GPL-3.0 license
Project home page: `https://github.com/lab-medvedeva/Article-GADES`
PID: swh:1:snp:48fb0f5536ca7ee4cd53156e0b5e7373f2c4af4e
Citation: Akhtyamov, P., Nabi,A., Gafurov,V., Sizykh,A., Favorov,A., Medvedeva,Y., Stupnikov,A. "Article-GADES" [Computer software]. Software Heritage, `https://archive.softwareheritage.org/swh:1:snp:48fb0f5536ca7ee4cd53156e0b5e7373f2c4af4e;origin=https://github.com/lab-medvedeva/Article-GADES`
Operating system(s): Platform independent
Programming language: bash, R 4.1+, Python 3.7+
Other requirements: see public environment file

GADES reproducibility workflow
License: GPL-v3
Link: `https://doi.org/10.48546/WORKFLOWHUB.WORKFLOW.1125.1`
PID: 10.48546/WORKFLOWHUB.WORKFLOW.1125.1
Citation: Akhtyamov, P. (2024). GADES reproducibility workflow. WorkflowHub. `https://doi.org/10.48546/WORKFLOWHUB.WORKFLOW.1125.1`

Availability of supporting data and materials

Simulated data
All the code for data download or simulation, extraction and pre-processing can be found at Article-*GADES*.

Public data
Publicly available datasets were analyzed in this study:

- PBMC3K: SRP073767
- PBMC3K B& T: SRP073767
- PBMC3K: B& CD8T: SRP073767
- HLCA marrow: GSE109774
- HLCA aorta: GSE109774
- HLCA lung: GSE109774
- HSC: GSE96769
- HumanCortex: GSE75140
- FibroCard ATAC: GSE165837
- FibroCard RNA: GSE165838
- MouseHypothalamus: GSE87544
- PBMC5K: GSE129785
- TCells: GSE107223
- CellLines: GSE65360

Additional files

Supplementary Figure 1. Benchmarking results for dense generated datasets and all the benchmarked packages. Log-scaled packages running time for different input matrix size $|W|$.

Supplementary Figure 2. Benchmarking results for sparse generated datasets and all GADES modes: the impact of the data sparsity degree on Log-scaled packages running time.

Supplementary Figure 3. Benchmarking results for dense generated datasets for batch size usage. Acceleration running time for different input matrix size $|W|$.

Supplementary Figure 4. Lineplots of the memory usage for the tested methods to the number of non-zero elements and the $|W|$. GADES-GPU-sparse memory usage is proportional to the number of non-zero elements, whereas the other methods are proportional to the number of elements.

Supplementary Table 1. Summary for simulated matrices used for benchmarking packages on dense data.

Supplementary Table 2. Summary for simulated matrices used for benchmarking packages on sparse data.

Supplementary Table 3. Summary for the real experimental datasets used for benchmarking.

Supplementary Table 4. Benchmarking results for dense generated datasets

Supplementary Table 5. Adjusted R^2 values for linear models of logarithmic memory usage to log $|W|$ and logarithmic non-zero elements respectively.

Declarations

List of abbreviations

- CPU - Central Processing Unit
- RAM - Random Access Memory
- CUDA - Compute Unified Device Architecture
- GPU - Graphical Processing Unit
- scATAC-seq - single cell ATAC sequencing
- scRNA-seq - single cell RNA sequencing
- GADES - GPU-Assisted Distance Matrix Estimation Software
- GADES-CPU-dense - GADES package run with CPU acceleration and no account for data sparsity
- GADES-GPU-dense - GADES package run with GPU acceleration and no account for data sparsity
- GADES-CPU-sparse - GADES package run with CPU acceleration and specific mode accounting for data sparsity
- GADES-GPU-sparse - GADES package run with GPU acceleration and specific mode accounting for data sparsity
- COO - Coordinate List format
- CSR - Compressed Sparse Row format

Consent for publication

Not applicable

Competing Interests

The authors declare they have no competing interests.

Funding

The study was partially supported by the Russian Science Foundation, grant number 22-74-00023 to Alexey Stupnikov.

Author's Contributions

Conceptualization was completed by Pavel Akhtyamov, Alexander Favorov, Alexey Stupnikov. Data curation was completed by Pavel Akhtyamov. Formal analysis was carried out by Pavel Akhtyamov, Ausaaf Nabi. Funding acquisition was found by Alexey Stupnikov. Investigation was completed by Pavel Akhtyamov, Alexey Stupnikov. Methodology was designed by Pavel Akhtyamov. Project administration was accomplished by Yulia Medvedeva, Alexey Stupnikov. Resources were provided by Yulia Medvedeva, Alexey Stupnikov. Software was created by Pavel Akhtyamov, Ausaaf Nabi, Vladislav Gafurov, Alexey Sizykh. Alexey Stupnikov supervised the project. Validation was completed by Pavel Akhtyamov, Ausaaf Nabi. Visualization was created by Pavel Akhtyamov, Ausaaf Nabi. Pavel Akhtyamov, Alexander Favorov, Yulia Medvedeva and Alexey Stupnikov ~~writed~~ wrote the original draft. Pavel Akhtyamov, Yulia Medvedeva, Alexey Stupnikov reviewed and edited the draft.

Potential implications

GADES can be effective in statistical inference of large or sparse datasets, such as single cell NGS datasets, social graph networks and stiffness matrices, and can be adapted for various types of analysis in bioinformatics, engineering, machine learning, and other fields.

References

1. Gorban AN, Zinovyev AY. Principal graphs and manifolds. In: Handbook of research on machine learning applications and trends: algorithms, methods, and techniques IGI Global; 2010.p. 28–59.
2. Wolf FA, Hamey FK, Plass M, Solana J, Dahlin JS, Göttgens B, et al. PAGA: graph abstraction reconciles clustering with trajectory inference through a topology preserving map of single cells. Genome biology 2019;20:1–9.
3. Kendall MG. A new measure of rank correlation. Biometrika 1938;30(1/2):81–93.
4. Chen L, Chen G, Wang F. Recommender systems based on user reviews: the state of the art. User Modeling and User-Adapted Interaction 2015;25:99–154.
5. Fkih F. Similarity measures for Collaborative Filtering-based Recommender Systems: Review and experimental comparison. Journal of King Saud University-Computer and Information Sciences 2022;34(9):7645–7669.
6. Lazar C, Taminau J, Meganck S, Steenhoff D, Coletta A, Molter C, et al. A survey on filter techniques for feature selection in gene expression microarray analysis. IEEE/ACM transactions on computational biology and bioinformatics 2012;9(4):1106–1119.
7. Meli R, Morris GM, Biggin PC. Scoring functions for protein-ligand binding affinity prediction using structure-based deep learning: A review. Frontiers in bioinformatics 2022;2:885983.
8. Lü L, Chen D, Ren XL, Zhang QM, Zhang YC, Zhou T. Vital nodes identification in complex networks. Physics reports 2016;650:1–63.
9. Qiao L, Zhang L, Chen S, Shen D. Data-driven graph construction and graph learning: A review. Neurocomputing 2018;312:336–351.
10. Gibbons JD, Kendall M. Rank correlation methods. Edward Arnold 1990;46.
11. Newson R. Parameters behind "nonparametric" statistics: Kendall's tau, Somers' D and median differences. The Stata Journal 2002;2(1):45–64.
12. Croux C, Dehon C. Influence functions of the Spearman and Kendall correlation measures. Statistical methods & applications 2010;19:497–515.

13. Amezquita RA, Lun AT, Becht E, Carey VJ, Carpp LN, Geistlinger L, et al. Orchestrating single-cell analysis with Bioconductor. Nature methods 2020;17(2):137–145.
14. Hicks SC, Townes FW, Teng M, Irizarry RA. Missing data and technical variability in single-cell RNA-sequencing experiments. Biostatistics 2018;19(4):562–578.
15. Andrews TS, Hemberg M. False signals induced by single-cell imputation. F1000Research 2018;7.
16. Hou W, Ji Z, Ji H, Hicks SC. A systematic evaluation of single-cell RNA-sequencing imputation methods. Genome biology 2020;21:1–30.
17. Akhtyamov P, Shaheen L, Raevskiy M, Stupnikov A, Medvedeva YA. scATAC-seq preprocessing and imputation evaluation system for visualization, clustering and digital footprinting. Briefings in Bioinformatics 2024;25(1):bbad447.
18. Bouland GA, Mahfouz A, Reinders MJ. Consequences and opportunities arising due to sparser single-cell RNA-seq datasets. Genome biology 2023;24(1):86.
19. Nolet CJ, Gala D, Raff E, Eaton J, Rees B, Zedlewski J, et al. GPU semiring primitives for sparse neighborhood methods. Proceedings of Machine Learning and Systems 2022;4:95–109.
20. Lucas A, Lucas MA, Biobase S, Package 'amap'; 2019.
21. Kassambara A. Practical guide to principal component methods in R: PCA, M (CA), FAMD, MFA, HCPC, factoextra, vol. 2. Sthda; 2017.
22. Virtanen P, Gommers R, Oliphant TE, Haberland M, Reddy T, Cournapeau D, et al. SciPy 1.0: fundamental algorithms for scientific computing in Python. Nature methods 2020;17(3):261–272.
23. McKinney W, et al. pandas: a foundational Python library for data analysis and statistics. Python for high performance and scientific computing 2011;14(9):1–9.
24. Ryabko BY. A fast on-line code. In: Doklady Akademii Nauk, vol. 306 Russian Academy of Sciences; 1989. p. 548–552.
25. Ryabko BY. A fast on-line adaptive code. IEEE transactions on information theory 1992;38(4):1400–1404.
26. Buluç A, Fineman JT, Frigo M, Gilbert JR, Leiserson CE. Parallel sparse matrix-vector and matrix-transpose-vector multiplication using compressed sparse blocks. In: Proceedings of the twenty-first annual symposium on Parallelism in algorithms and architectures; 2009. p. 233–244.
27. Zheng GX, Terry JM, Belgrader P, Ryvkin P, Bent ZW, Wilson R, et al. Massively parallel digital transcriptional profiling of single cells. Nature communications 2017;8(1):14049.
28. Schiller HB, Montoro DT, Simon LM, Rawlins EL, Meyer KB, Strunz M, et al. The human lung cell atlas: a high-resolution reference map of the human lung in health and disease. American journal of respiratory cell and molecular biology 2019;61(1):31–41.
29. Hocker JD, Poirion OB, Zhu F, Buchanan J, Zhang K, Chiou J, et al. Cardiac cell type–specific gene regulatory programs and disease risk association. Science advances 2021;7(20):eabf1444.
30. Camp JG, Badsha F, Florio M, Kanton S, Gerber T, Wilsch-Bräuninger M, et al. Human cerebral organoids recapitulate gene expression programs of fetal neocortex development. Proceedings of the National Academy of Sciences 2015;112(51):15672–15677.
31. Chen R, Wu X, Jiang L, Zhang Y. Single-cell RNA-seq reveals hypothalamic cell diversity. Cell reports 2017;18(13):3227–3241.
32. Buenrostro JD, Corces MR, Lareau CA, Wu B, Schep AN, Aryee MJ, et al. Integrated single-cell analysis maps the continuous regulatory landscape of human hematopoietic differentiation. Cell 2018;173(6):1535–1548.
33. Satpathy AT, Saligrama N, Buenrostro JD, Wei Y, Wu B, Rubin AJ, et al. Transcript-indexed ATAC-seq for precision immune profiling. Nature medicine 2018;24(5):580–590.
34. Buenrostro JD, Wu B, Litzenburger UM, Ruff D, Gonzales ML, Snyder MP, et al. Single-cell chromatin accessibility reveals principles of regulatory variation. Nature 2015;523(7561):486–490.
35. Waskom ML. Seaborn: statistical data visualization. Journal of Open Source Software 2021;6(60):3021.
36. Bengtsson H, profmem;. url: https://github.com/HenrikBengtsson/profmem.
37. Rodola Gea, psutil;. url: https://github.com/giampaolo/psutil.

Figure 1 Click here to access/download;Figure;Figure1.png

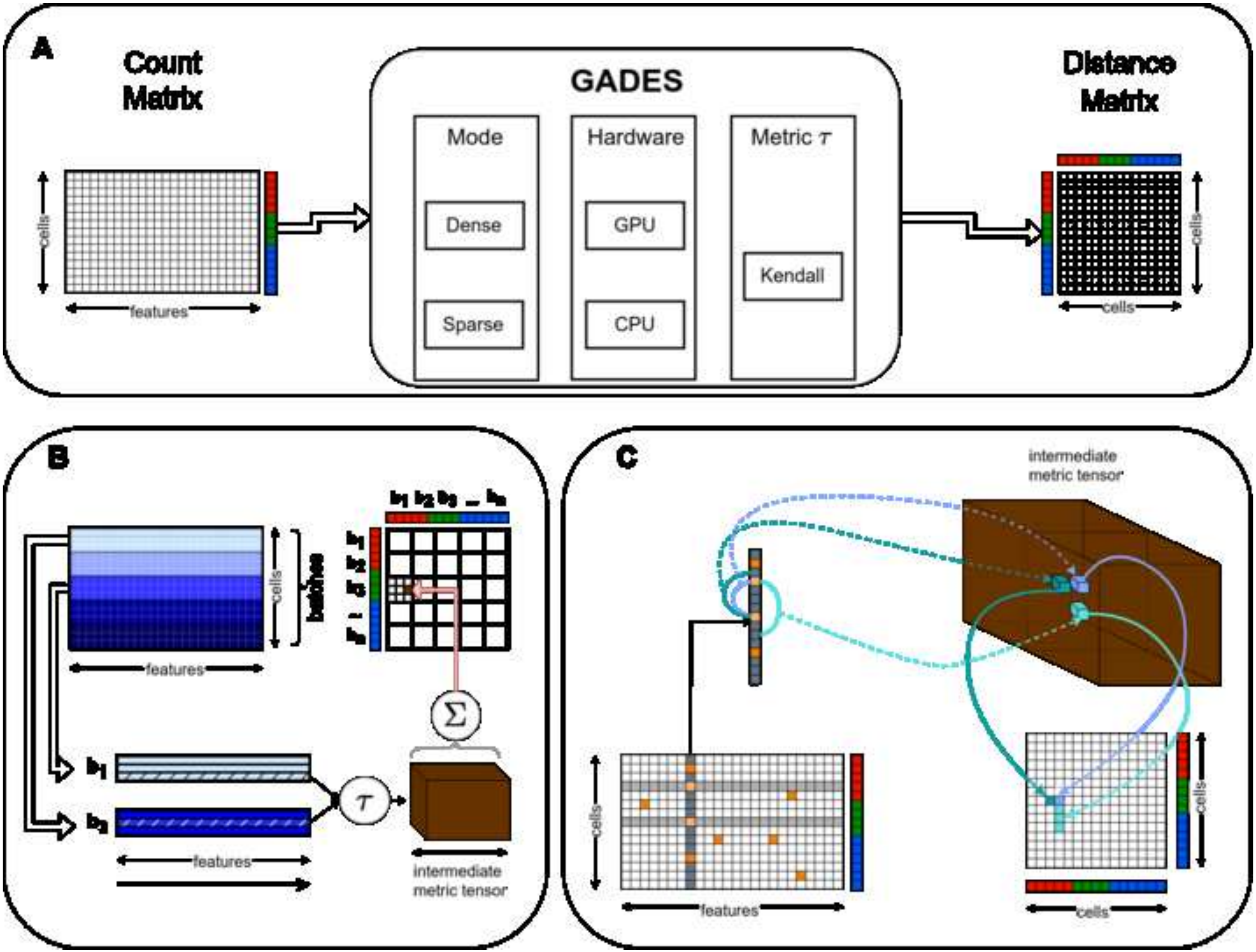

Figure 2

Click here to access/download;Figure;GadesAlgo.png

A

Algorithm 1: GADES algorithm for dense matrices

Data: Dense matrix M of size $C \times F$;
$Disc$ – discordant function for Kendall correlation
Result: DM of size $C \times C$

```
parallel for S = (u, d); 0 ≤ u ≤ d ≤ F − 1 do
    for 0 ≤ k < p < C do
        atomicAdd(DM_{k,p}, Disc(M_{k,S}, M_{p,S}));
    end
end
```

B

Algorithm 2: GADES algorithm for sparse matrices with Kendall distance

Data: Sparse matrix M of size $C \times F$: contains of $indices$, $values$ – lists of F elements;
$Disc$ – discordant function for Kendall correlation
Result: DM of size $C \times C$

```
parallel for S = (u, d); 0 ≤ u ≤ d ≤ F − 1 do
    col_count = # existing elements in M_{*,u};
    for 0 ≤ col1_index ≤ col2_index < col_count do
        l = indices[u][col1_index - 1];
        k = indices[u][col1_index];
        p = indices[u][col2_index];
        r = indices[u][col2_index + 1];
        M_{k,u} = values[u][col1_index];
        M_{p,u} = values[u][col2_index];
        move indices to range M_{a,d} for a ∈ (l, k) ∪ (p, r)
         with sweeping line;

        detect values of M_{a,S} for a ∈ (l, k) ∪ (p, r) with
         discovered indices;

        atomicAdd(DM_{k,p}, Disc(M_{k,S}, M_{p,S}));
        for l < a < k do
            atomicAdd(DM_{a,p}, Disc(M_{a,S}, M_{p,S}));
        end
        for p < a < r do
            atomicAdd(DM_{k,a}, Disc(M_{k,S}, M_{a,S}));
        end
    end
end
```

Figure 4

Click here to access/download;Figure;figure4.png

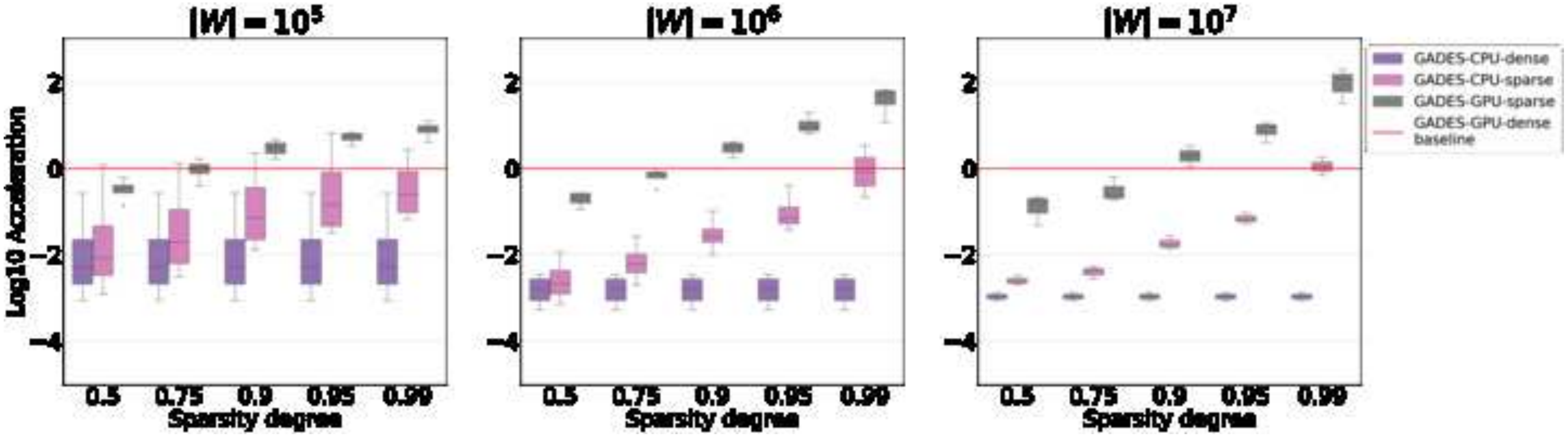

Figure 5

Click here to access/download;Figure;figure5.png

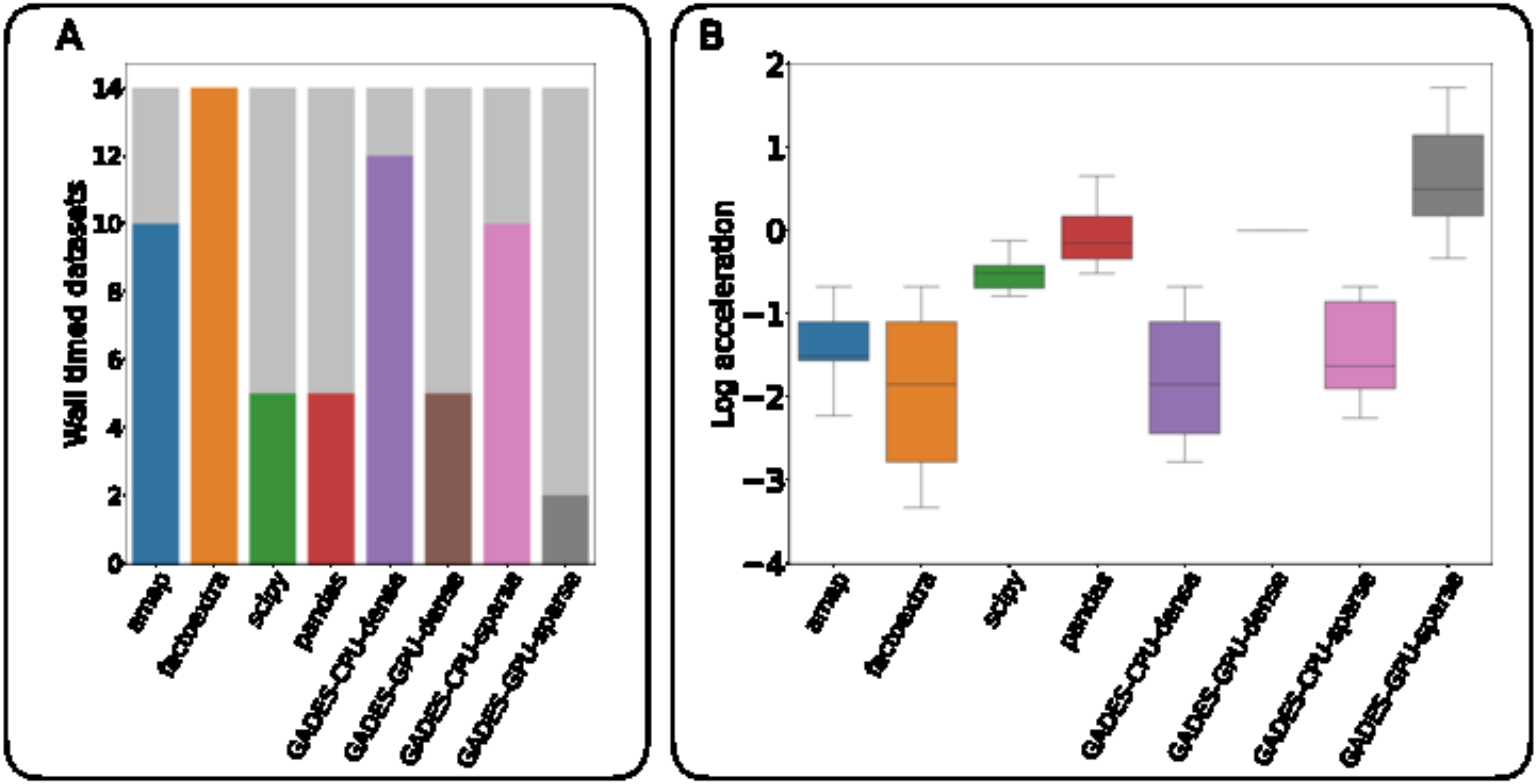

Figure 3 Click here to access/download;Figure;figure3.png

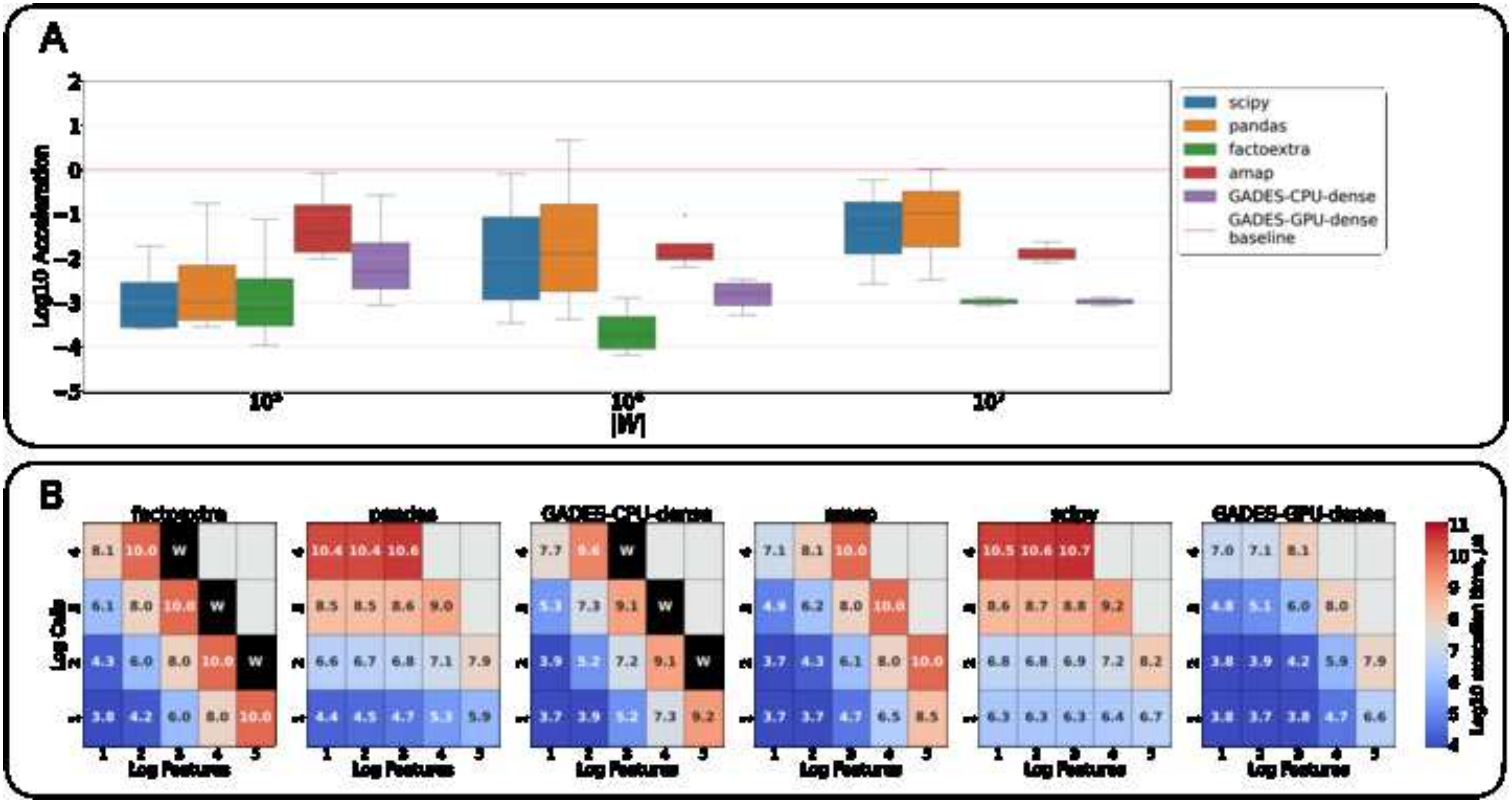

Figure 6

Click here to access/download;Figure;figure6.png

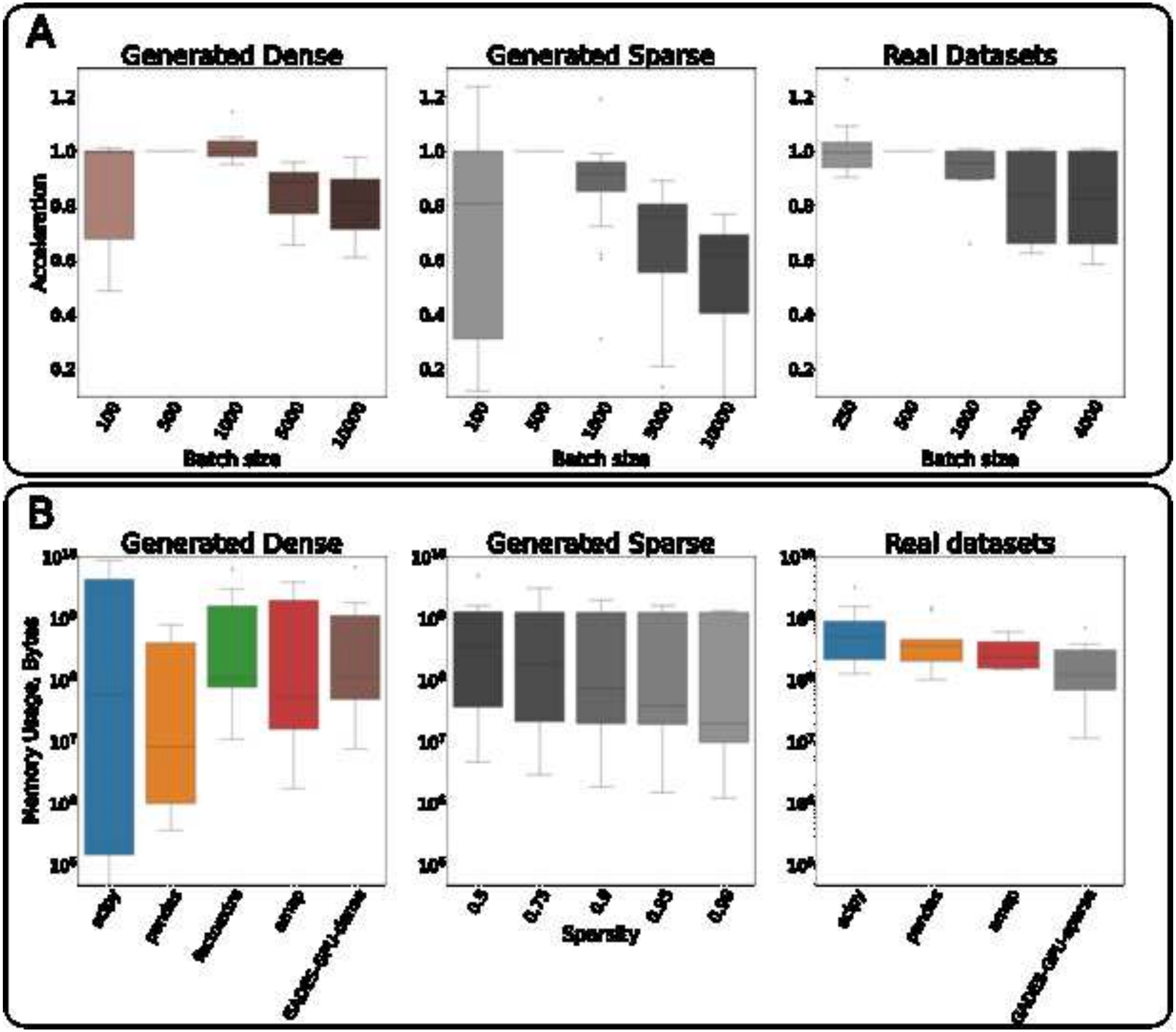

Click here to access/download

Supplementary Material

GADES-supplementary.pdf

Personal Cover Click here to access/download;Personal Cover;GigascienceCoverLetter.pdf

Dear Editor,

I am writing to submit our manuscript entitled: «GPU-accelerated Kendall distance computation for large or sparse data» to be considered for publication as a Technical Note in *GigaScience*.

Kendall distance matrices computation between samples is a commonly employed procedure for data preprocessing or statistical inference in numerous distinct research and industry fields. High dimensionality problem manifested with the beginning of Big Data era imposes sufficient computational challenges on data processing and manipulation procedures, including Kendall distance matrix generation for large datasets.

Recruitment of GPU to enhance the computational speed of distance matrices calculation appears to be a natural choice. Several CPU-based packages were implemented to compute Kendall distance matrices, however, as of today, no available package or software provides means for GPU-accelerated Kendall distance matrix generation.

Another avenue to enhance distance matrices computation efficiency is by taking advantage of commonly encountered data specific properties, such as sparsity. Although often considered a source of computational difficulties, sparsity-specific algorithms may assist with processing data more efficiently.

In our manuscript, we present GPU-Assisted Distance Estimation Software (GADES), a GPU-enhanced package that provides means for massively paralleled distance matrices computation with Kendal-τ metrics. Package's architecture incorporates specific memory management, which lifts the limits for the data size imposed by GPU memory capacity. Additional algorithmic solutions provide means to reinforce the acceleration effect for sparse datasets.

Benchmarking against available CPU-based packages on simulated and on real experimental single cell NGS datasets demonstrated significant increase in running speed of GADES compared to other methods in both sparse and dense data processing, with an additional performance boost for the sparse data.

Given the number of studies and applications making use of Kendall distance matrix computation and the lack of existing approaches for accelerating this analysis with the means of GPU, we believe this work can significantly contribute to the development of high-performance computational strategies and allows for the efficient processing of Big Data in distinct research and industry fields. We hope that this paper meets the general criteria of the *GigaScience* and that the information provided by the issue will find a proper audience among the readers of your Journal.

We confirm that neither the manuscript nor any parts of its content are currently under consideration or published in another journal. All authors have approved the manuscript and agree with its submission to *GigaScience.*

On behalf of all the authors
Sincerely yours,

Alexey Stupnikov
Laboratory for Bioinformatics of Cell Technology
Moscow Institute of Physics and Technology
Moscow, 141701, Russian Federation
aleksej.stupnikov@phystech.edu

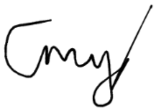

Supplement: giae088_GIGA-D-24-00103_Revisi3n_1 [file giae088_giga-d-24-00103_revisi3n_1.pdf]
