# Supplementary material for: GPU-accelerated Kendall distance computation for large or sparse data
Source: Gigascience. 2024 Dec 9;13:giae088. doi: 10.1093/gigascience/giae088 (PMC11631066; doi:10.1093/gigascience/giae088)
Supplement: giae088_Response_to_Reviewer_Comments_Original_Submission [file giae088_response_to_reviewer_comments_original_submission.pdf]

Author's Response To Reviewer Comments

Editor

In addition, please register any new software application in the bio.tools and SciCrunch.org databases to receive RRID (Research Resource Identification Initiative ID) and biotoolsID identifiers, and include these in your manuscript. Computational workflows should be registered in workflowhub.eu and the DOIs cited in the relevant places in the manuscript. These will facilitate tracking, reproducibility and re-use of your tool.

Following Editor's request we have registered the software in the listed databases and added the details to the Manuscript to the Availability of source code and requirements (Lines 457-458) and Availability of supporting data and materials (Lines 463-464) Sections

Reviewer 1
1. The manuscript should be carefully proofread, e.g., seems there are a few sentences or paragraphs missing in the front of the Introduction section.

Following the Reviewer's suggestion, we have extended the Introduction (Lines 1-14, 20-24, 28-33) section, and we thoroughly proofread and added minor corrections throughout the Manuscript.

2. The scientific contributions of this work should be strengthened.

Following the Reviewer's suggestion, we have added a paragraph on the work's potential significance in the Discussion section (Lines 415-425).

It would be more significant if the tool could support the computation of more distance metrics.

The Reviewer makes an excellent suggestion. Indeed, several popular distance metrics (such as Euclidean, Pearson, cosine. etc.) can also be accelerated with GPU implementation in general, and for some of those algorithmic implementations can be improved for highly sparse data, which can be useful in various practical applications. Their incorporation in the GADES package, therefore, does seem a natural strategy.

However, algorithmic approaches required by the Kendall metric and other metrics differ quite significantly. For optimal computational efficiency Pearson, Euclidean and cosine metrics, differently from Kendall metric, should be transduced to matrix multiplication, as GPU hardware is largely designed to multiply matrices to behalf on tensor cores with cuBLAS library. In this way, adapting GADES algorithm for these metrics with L2 cache technique (atomic operations), employed in GADES for Kendall distance computation, would work more slowly than an algorithm using the hardware-designed techniques. For this reason, the current version of GADES does not include these metrics.

These challenges can be resolved, yet require significant additional efforts in both software implementation and benchmarking. We agree with the Reviewer's suggestion and plan to incorporate their commendations, alongside other improvements, in the next versions of the package.

3. The configurations of the compared CPU-based methods should be provided. What is the effect of using more threads for these methods?

Following the Reviewer's suggestion, we have added the configurations for the cluster (Lines 223-224) and the CPU-based methods (Lines 227-236) to the manuscript.

4. It is helpful to report the memory usage of the compared methods.

The Reviewer poses an important question. Indeed the employed RAM resources, alongside running time, may potentially limit a package's efficiency. We have conducted the requested analysis and have added the corresponding subsections to the Methods (Lines 261-271), Results (Lines 361-379) and Discussion (Lines 407-414) sections.

5. The limitations of the tool should be discussed

Following the Reviewer's suggestion, we have expanded the paragraph on the method’s potential limitations in the Discussion section (Lines 426-436):

Reviewer 2

Weakness:

- Writing

1. The Kendall distance metric is not defined within this paper. This omission is particularly problematic when attempting to describe the algorithm, as it relies how the distance works.

Following the Reviewer's suggestion, we have added a paragraph on the distance metric mathematical definition in the Motivation section (Lines 52-58).

2. It is hard to read the exact data values from Figure 3. To enhance clarity, it would be beneficial to add a background grid to this figure or include a table providing the precise numerical values.

Following the Reviewer's suggestion, we have added background grids to the Figure 3A and 3B. Moreover, we added a separator between cells in the heatmap. Also, we added the requested table to the Supplementary file (Supplementary Table 4)

- Lacking of information

1. GADES also offers a CPU mode. However, it appears that this mode does not offer a significant advantage over other libraries. Therefore, why not consider offloading to these alternative libraries instead?

The Reviewer raises an important point. Indeed, the current version of GADES in CPU mode delivers no significant advantage over several existing CPU-based packages. This is largely due to the fact that the present implementation does not allow for multi-thread parallelization in GADES-CPU mode. The option for CPU parallelization is sought to be added in the next version of the package, and for this reason the offloading to other packages was not implemented.

2. The paper mentions the sparse matrix in GADES stores indices and values but doesn't explicitly specify the memory format. Is it the coordinate matrix format (COO) being used here?

We thank the Reviewer for pointing out the ambiguity of this point. We added the clarification in the

Methods section (Lines 153-156).

3. Would utilizing shared memory or alternative caching techniques be beneficial for performance improvement?

To address the Reviewer's question, we added the explanation in the Methods section (Lines 130-133):

4. The paper fails to analyze the impact of batch size on performance in the batch-wise processing step. Additionally, it does not report the specific batch size used in the experiments. This is a crucial parameter that influences both parallelism and the cost of atomic operations.

The Reviewer poses an important question. Indeed the batch size of the memory manipulation procedure may affect the algorithm’s running time, and an optimal batch size is required for the efficient practical use of the GADES package. We have conducted the requested analysis and have added the corresponding subsections to the Methods (Lines 247-260) and Results (Lines 344-360) sections.

5. Evaluation of Performance" section, if available, please provide details about the CPU configuration, including whether hyperthreading was enabled and if any virtualization technologies were employed on the HPC platform. Virtualization of CPU resources can sometimes lead to software spawning more threads than the allocated cores, potentially causing significant performance degradation

Following the Reviewer's suggestion, we have added the configurations for the cluster (Lines 223-224) and the CPU-based methods (Lines 227-236) to the manuscript.
