# Supplementary material for: GPU-accelerated Kendall distance computation for large or sparse data
Source: Gigascience. 2024 Dec 9;13:giae088. doi: 10.1093/gigascience/giae088 (PMC11631066; doi:10.1093/gigascience/giae088)
Supplement: giae088_Response_to_Reviewer_Comments_Revision_1 [file giae088_response_to_reviewer_comments_revision_1.pdf]

Author's Response To Reviewer Comments

Minor issues:
-Figure 3.A, Figure 6.B, and the second subfigure in Figure 6.B lacks the label of the x-axis.
It's recommended to add a paragraph after the first paragraph of the section Motivation (lines 58-59). The paragraph will explain why Kendall distance cannot be easily reduced to simple matrix multiplication and needs custom GPU acceleration code, and the importance of having a sparse mode. This will help explain the motivation.

Following the Reviewer's request, we have added the paragraph on the reasons, preventing us from simple matrix multiplication, in the Motivation section. Since we elaborate in the paragraph on GPU hardware-related algorithmic decisions, we felt the part of the section unpacking the practical challenges of GPU-based approach (lines 81-116) more fitting, and inserted the paragraph in this piece (lines 103-116).
