# Supplementary material for: GPU-accelerated Kendall distance computation for large or sparse data
Source: Gigascience. 2024 Dec 9;13:giae088. doi: 10.1093/gigascience/giae088 (PMC11631066; doi:10.1093/gigascience/giae088)
Supplement: giae088_Response_to_Reviewer_Comments_Revision_2 [file giae088_response_to_reviewer_comments_revision_2.pdf]

Author's Response To Reviewer Comments

Following the Editorial Team's request, we have altered the style of code and data references in the 'Code availability' and 'Data availability' sections
