# Supplementary material for: GPU-accelerated Kendall distance computation for large or sparse data
Source: Gigascience. 2024 Dec 9;13:giae088. doi: 10.1093/gigascience/giae088 (PMC11631066; doi:10.1093/gigascience/giae088)
Supplement: giae088_Reviewer_2_Report_Original_Submission — Huang Zhi-An -- 6/11/2024 [file giae088_reviewer_2_report_original_submission.pdf]

Reviewer Report

Title: GPU-accelerated Kendall distance computation for large or sparse data

Version: Original Submission **Date:** 6/11/2024

Reviewer name: Huang Zhi-An

Reviewer Comments to Author:

The paper presents GPU-Assisted Distance Estimation Software (GADES), a GPU-accelerated software for calculating the Kendall distance. The package provide considerable speed up in both dense and sparse cases compared to CPU-based implementation, and comes with an interface in the R language.Strength:1. The calculation is batching, thus having a good balance between performance and GPU memory usage.2. The author proposed two algorithms, one for dense matrix and one for sparse.3. A speedup in this operation can have a significant impact on the overall performance of certain real-life tasks.Weakness:- Writing1. The Kendall distance metric is not defined within this paper. This omission is particularly problematic when attempting to describe the algorithm, as it relies how the distance works.2. It is hard to read the exact data values from Figure 3. To enhance clarity, it would be beneficial to add a background grid to this figure or include a table providing the precise numerical values.- Lacking of information1. GADES also offers a CPU mode. However, it appears that this mode does not offer a significant advantage over other libraries. Therefore, why not consider offloading to these alternative libraries instead?2. The paper mentions the sparse matrix in GADES stores indices and values but doesn't explicitly specify the memory format. Is it the coordinate matrix format (COO) being used here?3. Would utilizing shared memory or alternative caching techniques be beneficial for performance improvement?4. The paper fails to analyze the impact of batch size on performance in the batch-wise processing step. Additionally, it does not report the specific batch size used in the experiments. This is a crucial parameter that influences both parallelism and the cost of atomic operations.5. Evaluation of Performance" section, if available, please provide details about the CPU configuration, including whether hyperthreading was enabled and if any virtualization technologies were employed on the HPC platform. Virtualization of CPU resources can sometimes lead to software spawning more threads than the allocated cores, potentially causing significant performance degradation.

Level of Interest

Please indicate how interesting you found the manuscript: Choose an item.

Quality of Written English

Please indicate the quality of language in the manuscript: Choose an item.

Declaration of Competing Interests

Please complete a declaration of competing interests, considering the following questions:

- Have you in the past five years received reimbursements, fees, funding, or salary from an organisation that may in any way gain or lose financially from the publication of this manuscript, either now or in the future?
- Do you hold any stocks or shares in an organisation that may in any way gain or lose financially from the publication of this manuscript, either now or in the future?
- Do you hold or are you currently applying for any patents relating to the content of the manuscript?
- Have you received reimbursements, fees, funding, or salary from an organization that holds or has applied for patents relating to the content of the manuscript?
- Do you have any other financial competing interests?
- Do you have any non-financial competing interests in relation to this paper?

If you can answer no to all of the above, write 'I declare that I have no competing interests' below. If your reply is yes to any, please give details below.

I declare that I have no competing interests

I agree to the open peer review policy of the journal. I understand that my name will be included on my report to the authors and, if the manuscript is accepted for publication, my named report including any attachments I upload will be posted on the website along with the authors' responses. I agree for my report to be made available under an Open Access Creative Commons CC-BY license (http://creativecommons.org/licenses/by/4.0/). I understand that any comments which I do not wish to be included in my named report can be included as confidential comments to the editors, which will not be published.

Choose an item.

To further support our reviewers, we have joined with Publons, where you can gain additional credit to further highlight your hard work (see: https://publons.com/journal/530/gigascience). On publication of this paper, your review will be automatically added to Publons, you can then choose whether or not to claim your Publons credit. I understand this statement.

Yes Choose an item.
